# Global burden of chronic kidney disease due to dietary factors

**DOI:** 10.3389/fnut.2024.1522555

**Published:** 2025-01-15

**Authors:** Lingtao Yin, Mengni Kuai, Zhuo Liu, Binbin Zou, Ping Wu

**Affiliations:** ^1^Department of Pharmacy, Loudi Hospital of Traditional Chinese Medicine, Loudi, Hunan, China; ^2^Department of Pharmacy, Changde Hospital, Xiangya School of Medicine, Central South University (The First People’s Hospital of Changde City), Changde, Hunan, China; ^3^College of Traditional Chinese Medicine, Changsha Medical University, Changsha, Hunan, China; ^4^Department of Hematology, Hunan Provincial People’s Hospital, The First Affiliated Hospital of Hunan Normal University, Changsha, Hunan, China

**Keywords:** chronic kidney disease, incidence rate, disability-adjusted life years, disease burden, adult

## Abstract

**Background:**

We aimed to assess the global impact of chronic kidney disease (CKD) attributable to dietary risk factors.

**Methods:**

The research utilized data from the Global Burden of Disease Study 2021 to evaluate age-standardized mortality rates (ASMR), disability-adjusted life years (DALYs), and estimated annual percentage changes (EAPCs) linked to CKD resulting from dietary risk factors.

**Results:**

From 1990 to 2021, both the ASMR and age-standardized DALY rate (ASDR) for CKD attributable to dietary risk factors exhibited an overall increasing trend globally. The mortality EAPC was 0.65, while the EAPC for DALYs stood at 0.39. Among dietary risk factors examined, a diet high in sugar-sweetened beverages was associated with the most substantial increase in CKD burden. Notably, Central sub-Saharan Africa bore the highest burden of CKD due to dietary risk factors, with an ASMR of 10.24 and an ASDR of 229.23. The increases in ASMR and ASDR were more pronounced in high-income regions, particularly in Latin America and the Caribbean, where the EAPC values for ASMR were 1.45 and 1.05, respectively, and for ASDR were 1.08 and 0.96. Furthermore, the burden of CKD was notably higher among middle-aged and elderly individuals, especially men aged 65 and above.

**Conclusion:**

The global disease burden attributed to dietary risk factors for CKD is increasing. A diet high in sugar-sweetened beverages exerted the most significant impact on CKD. There is a high incidence in Central sub-Saharan Africa, as well as in high-income regions and Latin America and the Caribbean.

## 1 Introduction

Chronic Kidney Disease (CKD) is an escalating global health issue. If left untreated, CKD can progressively worse, potentially leading to end-stage renal disease, necessitating dependence on dialysis or kidney transplantation (1). A 2023 study reports that the global prevalence of CKD stands at approximately 9.1%, with a rising trend, particularly in low- and middle-income countries where the issue is more pronounced (2). The two primary risk factors for CKD are hypertension and type 2 diabetes mellitus (3, 4). The incidence of both diabetes mellitus and hypertension is increasing exponentially and is anticipated to continue its upward trajectory in relation to CKD (5). The disability-adjusted life years of CKD are statistically forecasted to be the fifth most common cause of life expectancy loss by 2040 (6). However, the majority of cases are attributed to nutritional factors and are largely preventable, underscoring the critical importance of early prevention of CKD.

Dietary patterns represent a significant modifiable risk factor for CKD and are increasingly garnering attention (7). Research has demonstrated that adherence to healthy dietary habits is correlated with a reduced risk of CKD progression and overall mortality in patients with CKD (8). For instance, healthy dietary patterns such as the Dietary Approaches to Stop Hypertension (DASH) and Mediterranean diets (9), diets rich in whole grains (10), vegetables (11), and legumes (12), as well as lower consumption of red and processed meats (13), sodium (14), and sugar-sweetened beverages (15) have been linked to a decreased incidence of new-onset CKD and albuminuria. Furthermore, the selection of various nutrients and dietary patterns (16) can impact the progression of CKD in patients. Consequently, the investigation and promotion of healthy dietary patterns are crucial for the prevention and management of CKD.

Current comprehensive studies have examined the correlation between dietary patterns and the progression of CKD, focusing on disease progression deceleration and symptom alleviation through monitoring key nutrient intake, including protein, calcium, phosphorus, potassium, and sodium (17, 18). However, there is a paucity of research on the global burden of CKD attributable to dietary factors (19).

This study aims to provide an in-depth analysis of the specifics of CKD attributable to dietary risk factors and their global trends. It assesses data on the burden of CKD and the attributable dietary risk factors in 204 countries and territories, utilizing the GBD2021 database, a comprehensive and reliable source for assessing global health losses. The objective is to gain a profound understanding of the relationship between dietary risk factors and CKD, identify and quantify the contribution of these risk factors to the burden of CKD, and provide a scientific foundation for global dietary policy formulation and public health interventions.

## 2 Methods

### 2.1 Data sources

This study aims to conduct a comprehensive and systematic analysis of the global dynamics of disease burden attributable to dietary risk factors for CKD, based on the most recent research findings from the Global Burden of Disease 2021 (GBD 2021) database.^1^ The GBD project compiles and harmonizes data from a wide range of sources, including vital registration systems (such as death certificates), population-based surveys (e.g., household health surveys and nutrition surveys), disease registries, administrative data (hospital records, health insurance data), published literature (peer-reviewed articles, reports), and other relevant data repositories. By systematically analyzing and adjusting these data sources for biases and variations in data quality, the GBD provides comparable estimates of disease burden across different populations, time periods, and geographical regions. As a globally recognized health data platform, GBD 2021’s cause-of-death analysis encompasses 204 countries and regions, spanning the period from 1990 to 2021. It provides an in-depth analysis of mortality rates and years of life lost (YLLs) for 288 causes of death, disaggregated by age, sex, geographic location, and year. The dataset also includes information from 811 sub-administrative regions, ensuring a comprehensive analysis (20). In this study, annual statistics on CKD mortality, disability-adjusted life years (DALYs), and their corresponding age-standardized rates (ASRs) were extracted from the GBD 2021 dataset for the period 1990 to 2021. These statistics are accompanied by exhaustive 95% uncertainty intervals (UIs), which will be used to assess the role of dietary risk factors in this global health challenge.

The study identified cases of CKD attributable to dietary risk factors using the International Classification of Diseases, 10th Edition (ICD-10) and 9th Edition (ICD-9) codes. The ICD-10 codes utilized were N02-N02.9, N07-N07.9, Q60-Q63.2, Q63.8-Q63.9, and Q64.2-Q64.9, while the ICD-9 codes included 753.0–753.4 and 753.6–753.9. For the use of identified data in GBD study, a waiver of informed consent has been approved by the University of Washington Institutional Review Board. This study did not involve individual participants. The ethics approval can be found at https://www.healthdata.org/.

### 2.2 Estimates of the burden of CKD

In this research, we utilized DALY as the primary composite measure. DALYs are calculated as the sum of years of life lost (YLLs) due to premature mortality and years lived with disability (YLDs) (21). YLLs are derived by multiplying the number of deaths at each age by the remaining standard life expectancy at that age, while YLDs are calculated by multiplying the prevalence of a certain condition by a corresponding disability weight that reflects the severity of health loss. Furthermore, to accurately capture the influence of dietary risk factors on CKD’s disease burden, we employed the age-standardized mortality rate (ASMR) and age-standardized DALY rate (ASDR) as pivotal metrics. ASMR is computed by taking the total number of deaths from a given cause per 100,000 individuals in a population and adjusting for differences in the age structure using a standard population age distribution. Similarly, ASDR is calculated by taking the total DALYs due to a certain cause per 100,000 individuals, also standardized to a reference age structure. The age-standardization process ensures that observed differences in mortality or DALY rates between populations are not driven by differences in their age distributions, allowing for fair comparisons across countries, regions, and time periods.

### 2.3 Socio-demographic index

The Socio-demographic Index (SDI) is a composite measure that captures the overall socio-demographic development of a country or region. It is derived from a combination of key indicators, typically including average income per person (lag-distributed income), average educational attainment (mean years of schooling in the population aged 15 years and older), and total fertility rates under the age of 25. The SDI ranges from 0 to 1, with higher values reflecting greater socio-demographic development. This measure allows for analyzing how different socio-demographic conditions might influence disease burden and health outcomes globally.

### 2.4 Attributable dietary risk factors

Our research utilized DALYs related to CKD and corresponding data, employing dismod-mr 2.1 and spatio-temporal Gaussian process regression to quantify the influence of these risk factors on disease (22). This study encompassed seven dietary risk factors: diet low in fruits, diet low in vegetables, diet low in whole grains, diet high in red meat, diet high in processed meat, diet high in sugar-sweetened beverages, and diet high in sodium. All data on dietary risk factors were sourced directly from the GBD 2021 database, which integrates information from various national and international dietary surveys, food consumption databases, and nutritional studies. We adhered strictly to the GBD’s standardized definitions and thresholds for classifying diets as poor or excessive in specific food types, based on global dietary guidelines and expert consensus. Cultural differences and regional consumption patterns were inherently considered in the GBD’s data collection and modeling processes, ensuring that dietary risk factor classifications accurately reflect diverse dietary behaviors across different countries and regions.

### 2.5 Statistical analysis

In order to assess the disease burden stemming from dietary risk factors linked with CKD, we employed a directly standardized method to compute ASRs of mortality and DALY per 100,000 individuals, along with their respective 95% UIs. Furthermore, we utilized the estimated annual percentage change (EAPC) to quantify the temporal variation in age-standardized CKD mortality and DALY rates attributable to dietary risk factors over the period from 1990 to 2021. This was achieved by fitting a regression line to the natural logarithm of the rate (y = α + βx + ε), where y denotes the natural logarithm of the rate and x signifies the calendar year. The EAPC was derived by multiplying 100 by (exp[β]-1), and its 95% UI was obtained from a linear regression model (23). All statistical analyses conducted in this study were executed using R software (version 4.3.2), and *p* values below 0.05 were deemed statistically significant.

## 3 Results

### 3.1 Global trends in burden of CKD due to dietary risks factors

**Figure 1:**
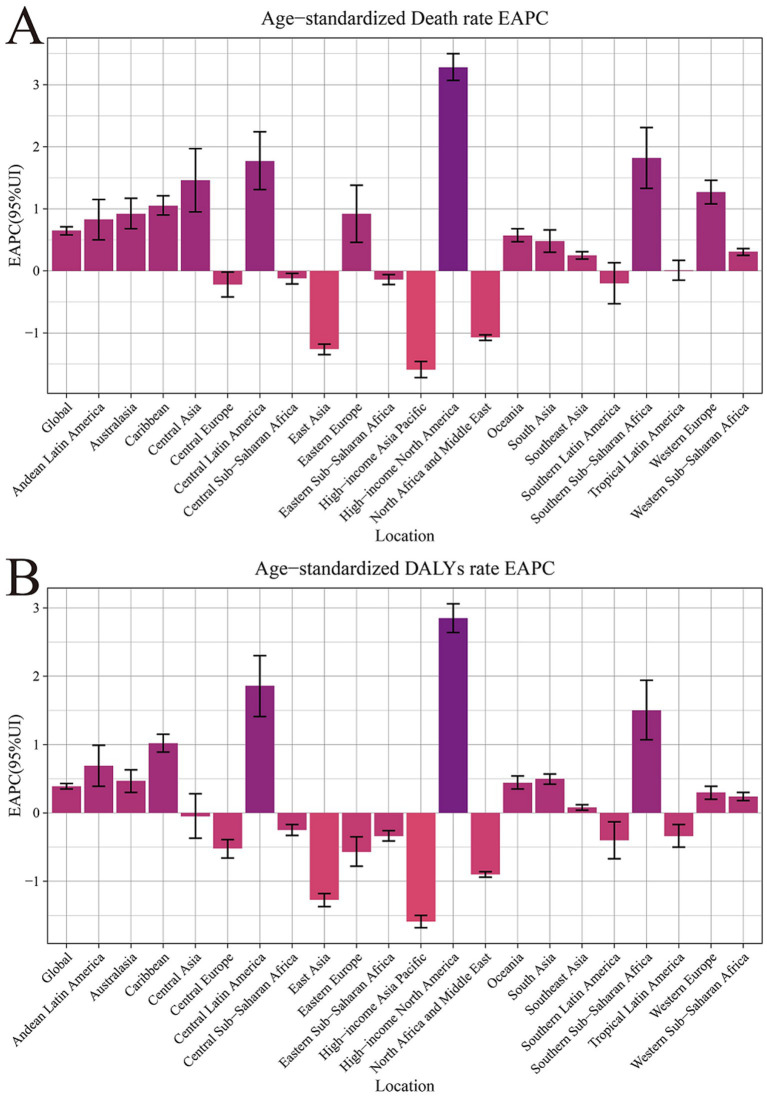
Age-standardized rates and estimated annual percentage change for chronic kidney disease due to dietary factors by 21 regions, from 1990 to 2021. **(A)** Age-standardized mortality rates (ASMR). **(B)** Age-standardized DALY rates (ASDR).

From 1990 to 2021, the age-standardized mortality rates for CKD attributable to dietary risk factors and the corresponding DALYs exhibited an increasing trend across 21 global regions. The EAPC in mortality rates was 0.65 (95% CI: 0.58 to 0.71), while the EAPC for DALYs stood at 0.39 (95% CI, 0.35 to 0.43). A detailed examination revealed that the ASMRs consistently rose from 3.23% in 1990 to 3.83% in 2021, marking a rise of 0.60 percentage points over three decades—a trend warranting attention. Concurrently, the age-standardized rate of DALYs demonstrated a varied upward trajectory, accumulating an increase of 9.4 percentage points since 1990, advancing from 84.12 to 93.52% (Table 1; Figure 1).

**Table 1:** Age-standardized mortality and DALY rates (ASMR and ASDR) and estimated annual percentage change (EAPC) for chronic kidney disease (CKD) attributable to dietary risk factors by super-region and region, 1990–2021.

	Age-standardized rates per 100,000 population (95% UI)				EAPC from 1990 to 2019 (95% CI)	
	1990	1990	2021	2021	1990	2021
	DALY rate	Death rate	DALY rate	Death rate	DALY rate	Death rate
Global						
both	84.12 (49.75, 120.66)	3.23 (1.92, 4.57)	93.52 (54.29, 134.38)	3.83 (2.25, 5.49)	0.39 (0.35, 0.43)	0.65 (0.58, 0.71)
female	72.60 (41.92, 102.87)	2.72 (1.63, 3.87)	80.69 (47.55, 116.70)	3.28 (1.94, 4.76)	0.36 (0.31, 0.41)	0.68 (0.60, 0.75)
male	98.95 (58.28, 142.31)	4.00 (2.35, 5.76)	108.58 (62.74, 159.15)	4.56 (2.67, 6.66)	0.37 (0.34, 0.41)	0.54 (0.49, 0.59)
Super_regions						
Central Europe, eastern Europe, and central Asia	52.80 (29.93, 77.29)	1.33 (0.74, 1.99)	50.82 (28.19, 75.79)	1.60 (0.87, 2.45)	−0.29 (−0.38, −0.20)	0.59 (0.47, 0.71)
High-income	56.30 (32.83, 80.75)	2.27 (1.37, 3.28)	72.66 (43.74, 101.75)	3.21 (1.87, 4.60)	1.08 (0.97, 1.19)	1.45 (1.33, 1.58)
Latin America and Caribbean	136.77 (84.54, 187.81)	5.61 (3.44, 7.76)	172.76 (104.57, 243.20)	7.10 (4.28, 10.13)	0.96 (0.70, 1.22)	1.05 (0.78, 1.33)
North Africa and Middle East	92.87 (50.62, 145.34)	4.32 (2.35, 6.98)	87.79 (49.91, 130.38)	4.11 (2.28, 6.09)	−0.90 (−0.94, −0.86)	−1.07 (−1.12, −1.03)
South Asia	92.75 (52.60, 138.03)	3.10 (1.76, 4.51)	105.16 (58.46, 160.45)	3.67 (1.96, 5.62)	0.50 (0.42, 0.57)	0.48 (0.30, 0.66)
Southeast Asia, east Asia, and Oceania	90.99 (51.72, 131.68)	3.80 (2.17, 5.59)	74.66 (42.13, 111.48)	3.14 (1.75, 4.70)	−0.66 (−0.73, −0.59)	−0.68 (−0.74, −0.62)
Sub-Saharan Africa	174.19 (98.63, 254.07)	7.75 (4.43, 11.34)	187.27 (106.65, 275.11)	8.75 (4.86, 12.97)	0.12 (0.06, 0.19)	0.28 (0.20, 0.35)
Regions						
Andean Latin America	153.17 (94.16, 213.04)	7.18 (4.31, 10.08)	191.06 (112.76, 273.42)	9.27 (5.38, 13.50)	0.69 (0.39, 0.99)	0.83 (0.50, 1.15)
Australasia	40.54 (23.24, 58.83)	1.81 (1.04, 2.62)	42.46 (24.39, 60.99)	2.03 (1.17, 2.88)	0.47 (0.30, 0.63)	0.92 (0.68, 1.17)
Caribbean	114.79 (71.68, 157.67)	4.61 (2.85, 6.37)	141.05 (87.18, 201.24)	5.65 (3.38, 8.11)	1.02 (0.89, 1.15)	1.05 (0.90, 1.21)
Central Asia	60.24 (33.09, 91.16)	0.91 (0.46, 1.45)	68.65 (36.41, 106.02)	1.75 (0.87, 2.84)	−0.05 (−0.37, 0.28)	1.46 (0.95, 1.97)
Central Europe	75.13 (41.02, 111.06)	2.67 (1.46, 3.99)	59.87 (32.57, 91.84)	2.30 (1.22, 3.60)	−0.52 (−0.66, −0.39)	−0.22 (−0.42, −0.02)
Central Latin America	144.20 (86.55, 203.25)	6.04 (3.54, 8.68)	224.86 (129.21, 327.66)	8.91 (5.06, 13.03)	1.86 (1.41, 2.30)	1.77 (1.31, 2.24)
Central Sub-Saharan Africa	232.44 (133.34, 343.60)	10.03 (5.64, 14.90)	229.23 (128.39, 350.76)	10.24 (5.54, 15.96)	−0.25 (−0.33, −0.17)	−0.12 (−0.21, −0.04)
East Asia	72.47 (41.13, 107.44)	3.06 (1.75, 4.54)	48.70 (25.79, 74.68)	2.11 (1.08, 3.28)	−1.27 (−1.37, −1.18)	−1.26 (−1.35, −1.18)
Eastern Europe	39.22 (22.20, 57.11)	0.69 (0.39, 1.03)	38.27 (21.62, 56.27)	1.04 (0.57, 1.57)	−0.57 (−0.78, −0.35)	0.92 (0.46, 1.38)
Eastern Sub-Saharan Africa	196.26 (109.00, 296.74)	8.98 (5.01, 13.59)	186.90 (105.39, 283.60)	9.07 (5.08, 13.90)	−0.34 (−0.41, −0.26)	−0.14 (−0.22, −0.06)
High-income Asia Pacific	74.49 (41.28, 111.42)	3.36 (1.85, 5.10)	46.81 (25.77, 70.53)	2.16 (1.17, 3.32)	−1.59 (−1.68, −1.50)	−1.59 (−1.72, −1.46)
High-income North America	58.04 (35.15, 82.18)	2.23 (1.40, 3.08)	125.15 (76.36, 172.48)	5.43 (3.29, 7.44)	2.85 (2.64, 3.06)	3.28 (3.07, 3.50)
North Africa and Middle East	92.87 (50.62, 145.34)	4.32 (2.35, 6.98)	87.79 (49.91, 130.38)	4.11 (2.28, 6.09)	−0.90 (−0.94, −0.86)	−1.07 (−1.12, −1.03)
Oceania	55.50 (29.74, 84.75)	2.08 (1.05, 3.28)	64.85 (35.61, 99.93)	2.56 (1.37, 3.96)	0.44 (0.35, 0.54)	0.57 (0.47, 0.68)
South Asia	92.75 (52.60, 138.03)	3.10 (1.76, 4.51)	105.16 (58.46, 160.45)	3.67 (1.96, 5.62)	0.50 (0.42, 0.57)	0.48 (0.30, 0.66)
Southeast Asia	152.30 (86.86, 218.30)	6.19 (3.58, 8.90)	157.82 (88.52, 235.07)	6.78 (3.86, 9.95)	0.08 (0.04, 0.12)	0.25 (0.19, 0.31)
Southern Latin America	125.07 (74.08, 176.07)	6.03 (3.60, 8.53)	101.68 (58.55, 148.07)	5.19 (2.94, 7.65)	−0.40 (−0.67, −0.13)	−0.20 (−0.53, 0.13)
Southern Sub-Saharan Africa	118.16 (65.44, 177.78)	4.57 (2.51, 6.86)	196.76 (110.55, 283.63)	8.49 (4.72, 12.24)	1.50 (1.07, 1.94)	1.82 (1.33, 2.31)
Tropical Latin America	132.91 (83.54, 180.64)	5.13 (3.30, 7.01)	124.37 (77.61, 168.66)	5.16 (3.22, 7.07)	−0.34 (−0.50, −0.17)	0.01 (−0.15, 0.17)
Western Europe	44.32 (25.40, 64.81)	1.71 (1.01, 2.51)	44.32 (25.37, 64.23)	2.09 (1.21, 3.11)	0.30 (0.20, 0.39)	1.27 (1.08, 1.46)
Western Sub-Saharan Africa	159.33 (88.06, 233.19)	7.28 (4.11, 10.74)	174.29 (96.00, 261.75)	8.17 (4.44, 12.31)	0.24 (0.18, 0.30)	0.31 (0.25, 0.36)

Among the various factors contributing to dietary risk, a diet high in sugar-sweetened beverages had a particularly significant impact on CKD, with a mortality EAPC of 2.17 (95% CI: 1.98 to 2.36). This was followed by an EAPC for DALYs of 2.14 (95% CI: 1.99 to 2.29), highlighting the severe threat that sugar-sweetened beverages pose to kidney health. Additionally, dietary habits such as high consumption of red meat and processed meat were also identified as high-risk factors. The former had EAPCs of 1.33 (95% CI: 1.20 to 1.46) and 1.07 (95% CI: 0.96 to 1.18) in mortality and DALYs respectively, while the latter had EAPCs of 1.23 (95% CI: 1.07 to 1.40) and 0.74 (95% CI: 0.60 to 0.88; Table 2).

**Table 2:** Global age-standardized mortality and DALY rates (ASMR and ASDR) and rate changes attributable to dietary risk factors for chronic kidney disease (CKD), 1990 and 2021.

	Age-standardized rates				EAPC from 1990 to 2019 (95% CI)	
	1990	1990	2021	2021		
Factors	DALY rate	Death rate	DALY rate	Death rate	DALY rate	Death rate
Dietary risks	84.12 (49.75, 120.66)	3.23 (1.92, 4.57)	93.52 (54.29, 134.38)	3.83 (2.25, 5.49)	0.39 (0.35, 0.43)	0.65 (0.58, 0.71)
Diet low in fruits	36.50 (18.65, 54.04)	1.37 (0.72, 2.04)	38.68 (20.15, 57.77)	1.53 (0.79, 2.31)	0.19 (0.15, 0.23)	0.41 (0.36, 0.47)
Diet low in vegetables	29.63 (14.18, 47.77)	1.15 (0.56, 1.85)	30.84 (14.80, 50.20)	1.27 (0.62, 2.06)	0.18 (0.14, 0.22)	0.43 (0.39, 0.48)
Diet low in whole grains	5.80 (1.44, 10.99)	0.23 (0.06, 0.41)	6.46 (1.59, 12.52)	0.27 (0.07, 0.50)	0.41 (0.33, 0.48)	0.58 (0.50, 0.66)
Diet high in red meat	4.20 (0.00, 9.28)	0.16 (0.00, 0.36)	5.50 (0.00, 12.01)	0.23 (0.00, 0.50)	1.07 (0.96, 1.18)	1.33 (1.20, 1.46)
Diet high in processed meat	4.98 (1.25, 9.27)	0.18 (0.05, 0.33)	5.95 (1.53, 11.00)	0.25 (0.06, 0.44)	0.74 (0.60, 0.88)	1.23 (1.07, 1.40)
Diet high in sugar-sweetened beverages	1.23 (0.57, 2.18)	0.04 (0.02, 0.08)	2.22 (1.02, 3.74)	0.08 (0.04, 0.14)	2.14 (1.99, 2.29)	2.17 (1.98, 2.36)
Diet high in sodium	18.55 (3.12, 47.36)	0.73 (0.11, 1.92)	19.81 (2.51, 54.57)	0.84 (0.09, 2.39)	0.23 (0.19, 0.26)	0.49 (0.45, 0.52)

### 3.2 Burden of CKD in different countries and regions

**Figure 2:**
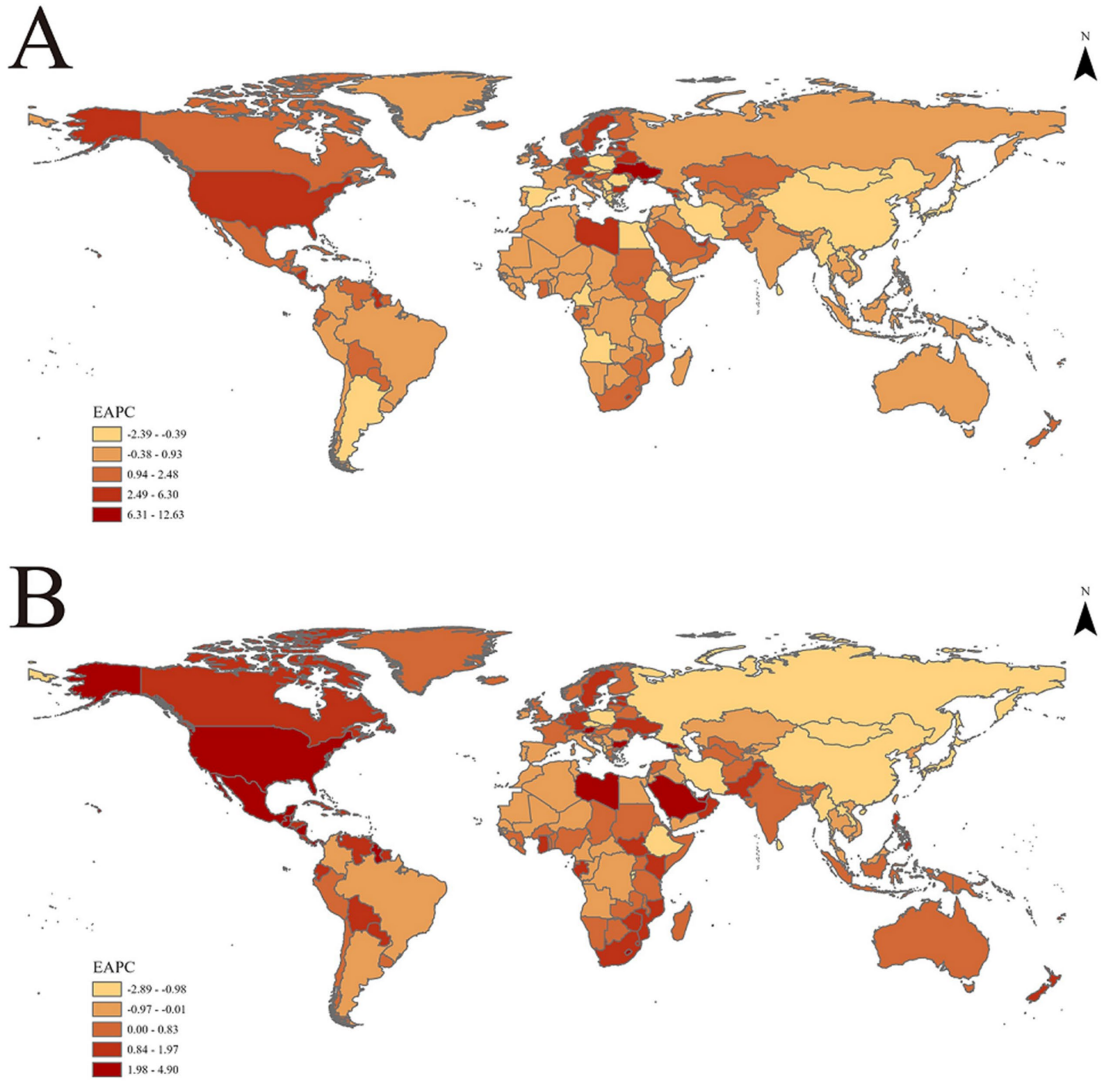
EAPC of age-standardized rates of chronic kidney disease due to dietary factors from 1990 to 2021, by locations. **(A)** Age-standardized mortality rates (ASMR). **(B)** Age-standardized DALY rates (ASDR).

Significant disparities exist in the ASMR and ASDR of CKD across various super regions and areas globally. The ASMR for five super regions, namely Central Europe, Eastern Europe, Central Asia, High Income, Latin America and the Caribbean, South Asia, and Sub-Saharan Africa, has demonstrated a consistent upward trend annually (EAPC>0). Notably, the High Income and Latin America and Caribbean regions exhibit particularly high EAPC values of 1.45 (95% CI: 1.33 to 1.58) and 1.05 (95% CI: 0.78 to 1.33) respectively, indicating a significant surge in CKD burden within these regions. Conversely, North Africa and the Middle East, Southeast Asia, East Asia, and Oceania have observed a downward trend in ASMR (EAPC<0). Southeast Asia, East Asia, and Oceania have shown the most substantial decline, with an EAPC value of −0.68 (95% CI: −0.74 to −0.62), suggesting that these regions have made considerable progress in CKD prevention and control(Table 1; Figures 1, 2).

In the trend of ASDR changes, regions such as High Income, Latin America and Caribbean, South Asia, and Sub-Saharan Africa demonstrated an upward trajectory. This was particularly noticeable in the High Income and Latin America and Caribbean regions, where the EAPC values for ASDR reached 1.08 (95% CI: 0.97 to 1.19) and 0.96 (95% CI: 0.70 to 1.22), respectively. Additionally, among the remaining 21 regions, 11 regions (including Andean Latin America, Australasia, Caribbean, etc.) exhibited an upward trend in both ASMR and ASDR. A particularly significant increase was observed in High Income North America, further underscoring the complexity and urgency of CKD prevention and control efforts worldwide (Table 1; Figures 1, 2).

Refined further at the national level, the United Arab Emirates is among the top countries in terms of increased ASMR and ASDR, with an EAPC value of 6.30 (95% Confidence Interval: 5.29 to 7.31) for ASMR and 4.90 (95% Confidence Interval: 4.13 to 5.67) for ASDR. This underscores the significant challenges the country faces in preventing and controlling CKD. Armenia and Georgia also rank highly in terms of rising ASMR, while Lesotho and American Samoa stand out in terms of increasing ASDR (Supplementary Table 1).

### 3.3 Burden and trends of CKD due to dietary risk factors in various countries and regions

From 1990 to 2021, the High income North America region exhibited the most pronounced increase in CKD mortality rates (EAPC = 3.28, 95% CI: 3.07 to 3.50) and DALYs (EAPC = 2.85, 95% CI: 2.64 to 3.06) attributed to various dietary risk factors globally. This suggests that high-income countries also grapple with addressing this public health challenge. Notably, the Central sub Saharan Africa region bears the greatest burden of CKD due to dietary risk factors. This is evident not only in its ASMR of 10.24 (95% UI: 5.54, 15.96), but also its leading position in ASDR with a rate of 229.23 (95% UI: 128.39, 350.76; Table 1; Figures 1, 2).

At the national level, Mauritius bears the most significant burden of CKD attributable to dietary risk factors, as evidenced by its high ASMR and ASDR. Conversely, Belarus exhibits the most favorable CKD ASMR, while Tajikistan demonstrates a comparatively lower ASDR. These findings offer potential benchmarks for the prevention and control of CKD in other nations (Supplementary Table 1).

Further examination of the influence of seven dietary risk factors on CKD mortality demonstrated that the Central sub-Saharan Africa region had a notably high CKD mortality rate, primarily due to diets low in fruits and vegetables. The ASMRs were 4.86 (95% UI: 2.44, 7.72) and 5.46 (95% UI: 2.58, 9.41), respectively. Conversely, the other five types of dietary risk factors - diets low in whole grains, high in red meat, high in processed meat, high in sugar-sweetened beverages, and high in sodium - resulted in higher CKD mortality rates in the Andean Latin America, Southern Latin America, High-income North America, Central Latin America, and Southeast Asia regions, respectively. In terms of ASDR, despite Central Latin America facing a high burden of CKD due to various dietary risk factors such as high sodium intake, high sugar-sweetened beverage consumption, and low vegetable intake, Central sub-Saharan Africa still holds a significant position in CKD high ASDR caused by factors such as low whole grain intake, high red meat consumption, and high processed meat consumption in Tropical Latin America. Additionally, the Southern Latin America region also faces a higher risk of CKD due to specific dietary risk factors (Supplementary Table 2).

### 3.4 Trends in burden of CKD due to dietary risk factors across genders and ages

**Figure 3:**
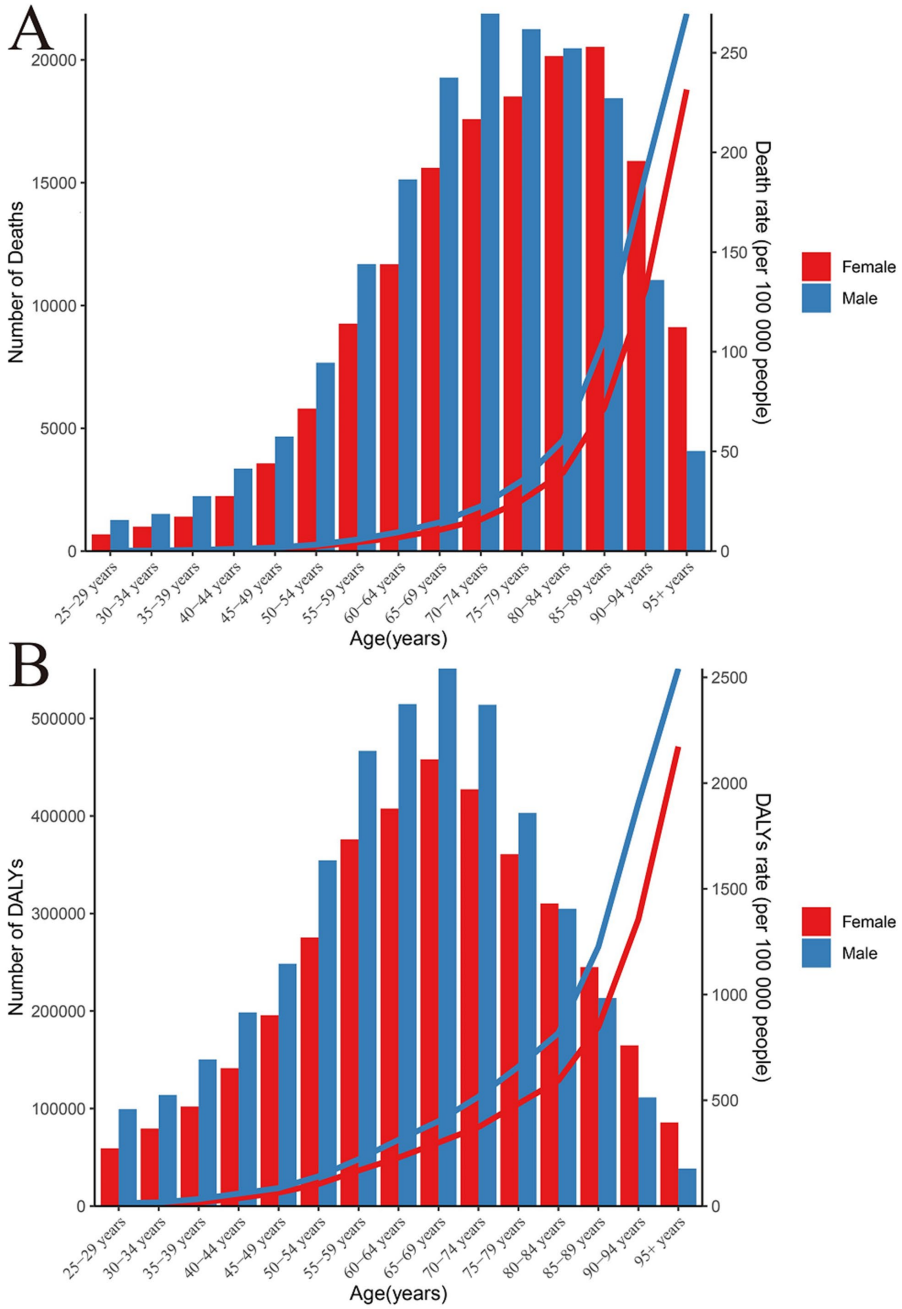
The mortality and DALY rates and number of chronic kidney disease due to dietary factors in different age groups. **(A)** Mortality rate and number. **(B)** DALY rate and number.

The impact of dietary risk factors on the prevalence of CKD is particularly pronounced among the elderly, especially men aged 65 and above. This suggests that the health risks associated with CKD, attributable to dietary risk factors, escalate with age. In 2021, there was a marked increase in CKD deaths caused by dietary risk factors among women, starting from the 45–49 age group, which continued to rise until the 90–94 age group, followed by a decline in the oldest age group. Conversely, men experienced this threat earlier, with an increase in deaths from the 40–44 age group. However, it is important to note that after reaching the 75–79 age group, the death toll began to gradually decline. Further analysis of DALYs indicators reveals a sharp increase in DALYs among females within the 40–69 age range, followed by a gradual decrease, while among males, DALYs continued to accumulate within the 35–69 age range before showing a downward trend. Additionally, the global disease burden is lowest among individuals aged 25–29, and highest among those aged 65 and above (Figure 3).

### 3.5 The relationship between SDI and burden of CKD disease due to dietary risk factors

**Figure 4:**
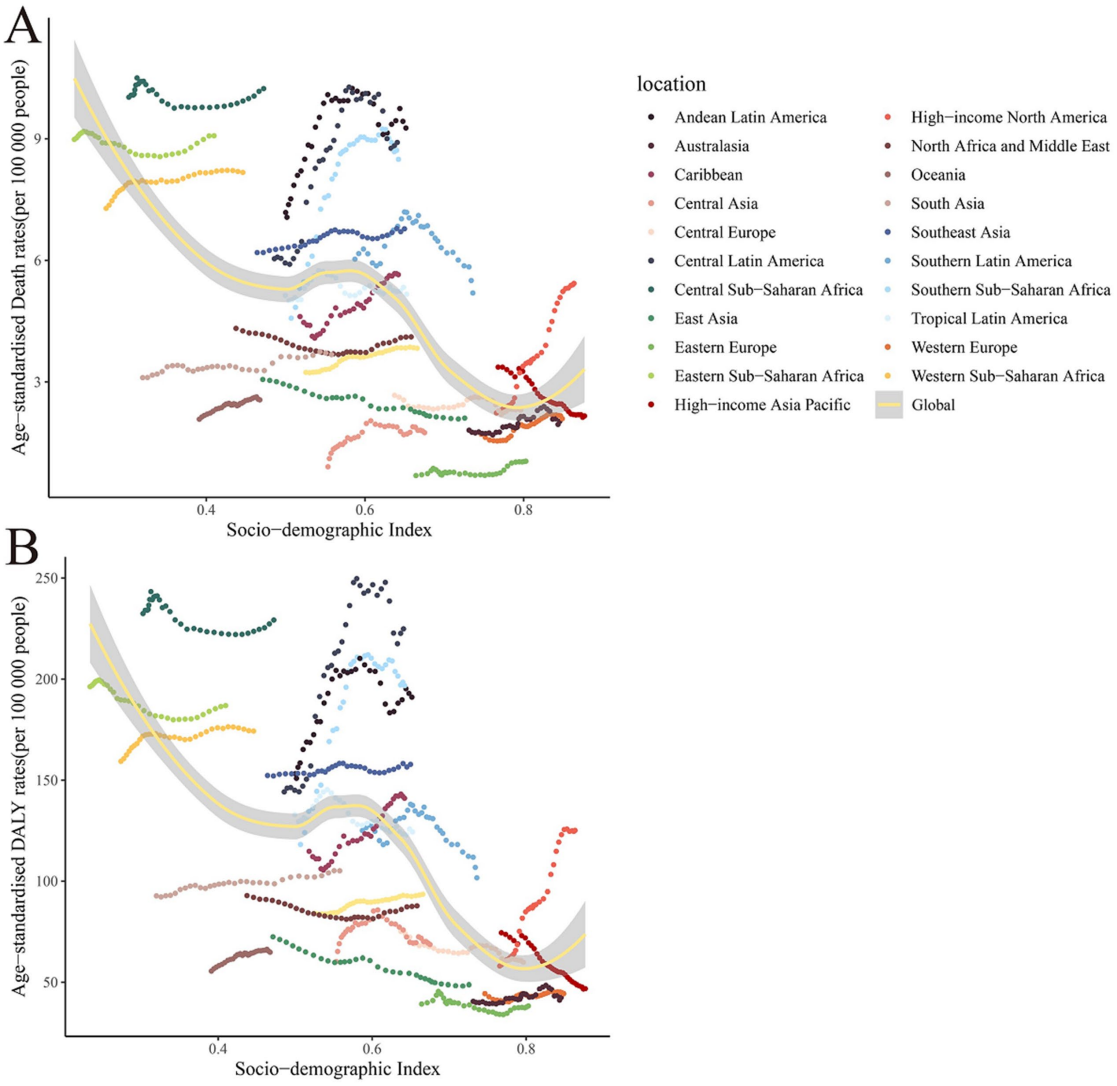
The relationship between age-standardized rates and SDI of global burden of chronic kidney disease due to dietary factors, by 21 regions. **(A)** Age-standardized mortality rates (ASMR). **(B)** Age-standardized DALY rates (ASDR).

After a comprehensive analysis of the correlation between demographic indices and CKD ASR across 204 countries and regions, we observed that CKD ASMR attributable to dietary risk factors demonstrated significant regional disparities globally (Figure 4). Specifically, Central Asia, Eastern Europe, and High-income Asia Pacific regions were among the top three in ASMR rankings. Conversely, North Africa and the Middle East, Southeast Asia, and Western Europe showed lower levels of CKD ASMR. Further examination of ASDR rankings revealed that Western sub-Saharan Africa, Tropical Latin America, and Eastern sub-Saharan Africa were among the top three regions, underscoring the substantial burden of CKD-related health risks in these areas.

## 4 Discussion

The global disease burden of CKD attributable to dietary risk factors has shown an upward trend, as indicated by increases in both ASMR and ASDR. This observed trend may be factors such as the prevalence of unhealthy dietary habits, an aging population, socio-economic development, lifestyle changes, and unequal access to healthcare resources (24, 25). Among the dietary risk factors examined, a diet high in sugar-sweetened beverages was associated with the most substantial increase in CKD burden. The burden of CKD varies significantly across countries and regions, with the highest burden attributable to dietary risk factors observed in Central sub-Saharan Africa and Mauritius. Additionally, the increase in ASMR and ASDR was more pronounced in high-income regions, as well as in Latin America, and the Caribbean. The burden of CKD was particularly higher among middle-aged and elderly individuals, especially men aged 65 years and older. These findings suggest the need for targeted public health policies and interventions to mitigate the rising burden of CKD associated with dietary risk factors.

Research has demonstrated a significant correlation between sugar-sweetened beverages (SASB) intake and CKD. Meta-analyses have confirmed this positive association, indicating that the risk of CKD in populations with high SBS consumption is 1.30 times greater than in low-consuming groups (26, 27). The potential for SASB to increase CKD risk may be attributed to several mechanisms, including body weight gain, altered blood glucose levels, elevated urate concentrations, and increased dietary phosphorus acid load (28). Furthermore, SASB consumption elevates the risk of diabetes, a primary risk factor for CKD. Persistently high blood glucose levels can overburden the kidney’s filtration system, leading to renal damage (29). Fructose metabolism in high-sugar beverages places stress on the liver and kidneys, and sustained high intake can result in insulin resistance, a precursor to diabetes (30). Concurrently, high-sugar beverages may cause elevated uric acid levels in the body, increasing the risk of gout and kidney damage (29). These beverages often contain artificial colors and additives, which may impair kidney function with prolonged intake (31). Therefore, reducing high-sugar beverage consumption is a crucial strategy for CKD prevention and management.

The prevalence of CKD attributable to dietary risk factors is notably high in Central sub-Saharan Africa and Mauritius. This can be attributed to a dietary and nutritional transition, propelled by rapid urbanization, economic development, and globalization. This transition has precipitated a dual burden of over-nutrition and under-nutrition, as well as unhealthy dietary habits such as insufficient intake of fruits, vegetables, grains, and an overconsumption of fatty foods high in red meat. These dietary habits escalate the risk of disability and mortality from CKD. Conversely, high fiber foods like grains, legumes, fruits, and vegetables mitigate renal load and inflammation, thereby decelerating the loss of renal function (32). In addition, malnutrition, driven by poverty, social injustice, and poor sanitation in these regions, may compromise immunity and increase susceptibility to various diseases, thereby impeding healthy physical development. In Central sub-Saharan Africa and Mauritius, the public healthcare system grapples with limited resources and extended waiting times, potentially resulting in diagnostic and treatment delays. Furthermore, Mauritius exhibits a high prevalence of non-communicable diseases (NCDs), particularly marked by elevated ASMR and DALY for CKD due to type 2 diabetes (33). The widespread occurrence of diabetes significantly contributes to the high burden of CKD. Therefore, targeted and comprehensive prevention and control strategies—including improved dietary habits, chronic disease management, enhanced accessibility to healthcare resources, and health education—are imperative to alleviate the burden of CKD.

Poor dietary habits in high-income and Latin America and Caribbean regions significantly contribute to the increased burden of CKD. The diets in these regions are characterized by a high intake of red meat, animal fats, sugar, and highly processed foods, while the consumption of vegetables and fruits is low. Such a Western-like dietary pattern has been linked to elevated levels of inflammation (34). In Latin America and the Caribbean, poor dietary habits primarily involve excessive consumption of high-salt foods and insufficient intake of vegetables and fruits, can elevate blood pressure. and chronic hypertension is a major risk factor for CKD (35), thereby increasing the burden on the kidneys. Furthermore, the consumption of high-sugar and highly processed foods is associated with an increased risk of obesity, diabetes, and CKD (36). The high sugar content and artificial additives in these foods pose a threat to kidney health. Red and processed meats contain high levels of sodium and potential carcinogens, and their excessive intake may lead to overnutrition or undernutrition, further increasing the risk of CKD (37). A significant portion of the population in Latin America and the Caribbean cannot afford a healthy diet due to economic constraints. Leading to hunger, childhood chronic malnutrition, and a high prevalence of non-communicable diseases, indirectly increase the CKD burden. Additional factors that exacerbate the risk of CKD include a lack of adequate physical activity, biased nutritional choices due to socioeconomic factors, and inadequate health awareness (32, 35). Therefore, improving dietary habits, reducing the intake of high-salt, sugar, and fat foods, and promoting healthy eating patterns are essential to reduce the burden of CKD.

The burden of CKD associated with dietary factors is notably pronounced in middle-aged and older adults, particularly men aged 65 and above. This increased susceptibility can be attributed to the natural decline in renal function as one ages (38). Furthermore, due to diminishing resistance, older adults frequently present with multiple comorbid chronic diseases such as diabetes and hypertension, which are significant risk factors for CKD. Concurrently, this demographic may encounter challenges in accessing healthcare resources and exhibit low awareness regarding CKD. Such barriers can hinder their ability to receive appropriate CKD treatment and might contribute to delays in early diagnosis and intervention. Biological and metabolic distinctions, coupled with a higher prevalence of chronic disease risk factors, may render men more susceptible to CKD (39). Specifically, diets high in protein and fat exacerbate this risk. Consequently, CKD prevention and management strategies must prioritize the middle-aged and older male population.

A correlation exists between the SDI and both the ASMR and ASDR of CKD attributable to dietary factors. As the SDI escalates, the trend in ASMR for CKD linked to dietary elements generally diminishes, with the lowest ASMR and ASDR observed in regions with a high SDI, and the highest in areas with a low SDI. This pattern may be attributed to superior medical resources and heightened health awareness in countries with a high SDI, facilitating earlier diagnosis and intervention for CKD, thereby reducing mortality rates and DALYs (2, 25). Conversely, both CKD mortality and DALYs are elevated in nations with a low SDI.

While our study highlights significant associations between dietary risk factors and the burden of CKD, it is important to acknowledge several limitations inherent in our analysis. Firstly, the observational nature of the study limits our ability to infer causality between dietary factors and CKD. The associations identified may be influenced by unmeasured confounding variables such as genetic predispositions, physical activity levels, socioeconomic status, and access to healthcare, which were not directly accounted for in our analysis. Secondly, the reliance on secondary data from the GBD 2021 database introduces potential biases related to data quality and availability, which can vary significantly across regions. Although the GBD employs rigorous statistical methods to adjust for these biases, some residual confounding and data inaccuracies may still affect the estimates. Additionally, accurately assessing dietary consumption patterns is inherently challenging due to variations in dietary assessment methods, reporting accuracy, and cultural differences in food consumption. The grouping of food types into broad categories may obscure specific interactions or effects of individual dietary components on CKD risk. Furthermore, certain regions may have limited data availability, leading to greater uncertainty in the estimates for those areas. Future research should aim to incorporate more granular dietary data, utilize longitudinal study designs, and account for a broader range of confounding factors to enhance the precision and validity of the associations between dietary risk factors and CKD.

In assessing the global burden of CKD disease attributable to dietary risk factors from 1990 to 2021, this study presents both strengths and limitations. Primarily, the study’s reliance on the GBD database means its accuracy is contingent upon the quality of the underlying data. This is particularly problematic in developing nations where data scarcity may introduce information bias. Additionally, CKD risk factors are multifaceted, encompassing metabolic, behavioral, and environmental dimensions. The intricate interplay of these factors might not be fully represented in current datasets, complicating the analytical process. Furthermore, while the study documents temporal trends in CKD disease burden, such trends can be shaped by a myriad of influences, including socioeconomic shifts, medical technological advancements, and lifestyle modifications, thereby complicating trend interpretation. Consequently, it is imperative to exercise caution when interpreting the findings, bearing in mind the inherent biases and uncertainties, to achieve a more nuanced understanding of CKD’s disease burden.

## 5 Conclusion

The global burden of CKD attributed to dietary risk factors is on the rise, with notable disparities observed across various countries and regions. The pronounced CKD burden in Central sub-Saharan Africa, coupled with the rising trend in high-income regions and Latin America and the Caribbean, underscores the need for tailored interventions. Moreover, the high-risk demographic of men aged 65 years further emphasizes the urgency for such preventive measures. Such a trend necessitates the implementation of targeted and comprehensive prevention and control strategies. These include rectifying poor dietary habits, advocating for healthy eating patterns to manage chronic diseases, and enhancing access to healthcare resources and health education.

## Data Availability

Publicly available datasets were analyzed in this study. This data can be found here: Global Health Data Exchange database (GHDx) (http://ghdx.healthdata.org/gbd-results-tool).

## Footnotes

^1^ https://vizhub.healthdata.org/gbd-results/
