# Supplementary material for: Global burden of chronic kidney disease due to dietary factors
Source: Front Nutr. 2025 Jan 15;11:1522555. doi: 10.3389/fnut.2024.1522555 (PMC11774714; doi:10.3389/fnut.2024.1522555)
Supplement: Supplementary file 1 [file Supplementary_file_1.doc]

Supplementary Table 1. Age-Standardized Mortality and DALY Rates (ASMR and ASDR) and Estimated Annual Percentage Change (EAPC) for Chronic Kidney Disease (CKD) Attributable to Dietary Risk Factors by Country, 1990–2021

|  | DALYs (Disability-Adjusted Life Years) | Deaths | DALYs (Disability-Adjusted Life Years) | Deaths | DALYs (Disability-Adjusted Life Years) | Deaths |
| --- | --- | --- | --- | --- | --- | --- |
|  | Age-standardized rate per 100 000 population (95% UI) | | Age-standardized rate per 100 000 population (95% UI) | | Estimated annual percentage change from 1990 to 2019 (95% CI) | |
|  | 1990 | 1990 | 2021 | 2021 |  |  |
|  | DALY rate | Death rate | DALY rate | Death rate | DALY rate | Death rate |
| Afghanistan | 248.32(129.71,404.84) | 10.44(5.29,17.88) | 266.89(122.52,497.10) | 11.79(5.49,21.97) | 0.25(0.20,0.30) | 0.45(0.39,0.51) |
| Albania | 86.82(47.58,133.02) | 3.31(1.76,5.33) | 53.88(25.46,93.15) | 2.21(1.00,4.04) | -1.56(-1.85,-1.27) | -1.23(-1.58,-0.87) |
| Algeria | 105.63(53.88,182.04) | 5.52(2.70,10.19) | 100.50(55.25,150.84) | 5.83(3.13,8.94) | -0.23(-0.42,-0.04) | 0.38(0.15,0.61) |
| American Samoa | 82.42(39.85,131.04) | 3.35(1.58,5.51) | 202.62(90.45,345.37) | 9.21(4.03,15.52) | 3.11(2.83,3.38) | 3.45(3.14,3.77) |
| Andorra | 54.26(29.18,83.74) | 2.53(1.33,4.27) | 41.61(22.79,61.48) | 1.78(0.94,2.74) | -0.64(-0.82,-0.45) | -0.76(-1.02,-0.50) |
| Angola | 208.16(109.57,317.88) | 8.81(4.70,13.44) | 185.76(98.44,295.25) | 8.55(4.63,13.69) | -0.67(-0.80,-0.54) | -0.40(-0.54,-0.26) |
| Antigua and Barbuda | 164.99(104.08,225.42) | 6.97(4.26,9.50) | 223.01(137.61,308.72) | 10.44(6.17,14.69) | 1.64(1.37,1.91) | 2.00(1.71,2.29) |
| Argentina | 142.71(85.79,201.68) | 6.96(4.15,9.82) | 111.12(63.68,160.94) | 5.58(3.18,8.12) | -0.56(-0.81,-0.30) | -0.45(-0.78,-0.12) |
| Armenia | 27.62(13.77,43.96) | 0.14(0.06,0.23) | 39.66(17.89,70.60) | 1.08(0.44,2.05) | 0.78(0.35,1.21) | 5.70(4.35,7.07) |
| Australia | 40.67(23.13,59.31) | 1.85(1.06,2.69) | 40.98(23.17,58.87) | 1.99(1.13,2.83) | 0.36(0.17,0.55) | 0.83(0.56,1.10) |
| Austria | 40.30(22.71,59.25) | 1.42(0.76,2.14) | 58.55(33.60,86.60) | 3.26(1.78,4.89) | 2.30(1.89,2.72) | 4.56(3.84,5.29) |
| Azerbaijan | 56.05(29.34,86.48) | 0.97(0.45,1.64) | 50.22(25.56,81.34) | 1.16(0.51,2.08) | -0.84(-1.12,-0.56) | 0.40(0.04,0.76) |
| Bahamas | 136.57(87.07,186.87) | 5.09(3.24,6.96) | 199.00(121.43,289.41) | 7.97(4.75,11.69) | 1.64(1.45,1.83) | 1.94(1.69,2.19) |
| Bahrain | 93.46(48.70,142.84) | 4.88(2.40,7.74) | 98.62(55.23,154.61) | 5.65(3.10,8.85) | -0.29(-0.55,-0.03) | 0.11(-0.22,0.44) |
| Bangladesh | 84.41(47.41,126.83) | 2.95(1.72,4.47) | 76.53(41.25,117.89) | 2.89(1.55,4.47) | -0.13(-0.26,-0.01) | -0.05(-0.29,0.18) |
| Barbados | 140.15(90.86,184.30) | 5.68(3.60,7.61) | 172.48(102.05,251.71) | 7.70(4.56,11.40) | 1.18(0.93,1.42) | 1.53(1.22,1.83) |
| Belarus | 22.28(12.04,34.02) | 0.14(0.07,0.21) | 24.90(13.85,37.81) | 0.36(0.20,0.56) | 0.07(-0.27,0.42) | 2.64(1.68,3.61) |
| Belgium | 52.77(30.02,77.94) | 2.12(1.23,3.13) | 47.70(27.09,71.59) | 2.05(1.11,3.15) | 0.19(0.01,0.37) | 0.51(0.25,0.78) |
| Belize | 123.38(76.07,169.52) | 5.00(3.07,7.05) | 210.01(127.83,294.41) | 8.62(5.13,12.26) | 2.25(1.84,2.66) | 2.29(1.81,2.77) |
| Benin | 175.88(92.32,261.30) | 8.31(4.53,12.38) | 183.07(98.11,283.26) | 8.85(4.69,13.64) | 0.05(-0.05,0.16) | 0.13(0.03,0.23) |
| Bermuda | 83.25(51.73,115.53) | 3.61(2.23,5.11) | 88.57(54.14,126.88) | 3.96(2.39,5.75) | 0.71(0.46,0.97) | 0.85(0.55,1.15) |
| Bhutan | 108.59(57.02,167.38) | 3.86(2.00,6.26) | 112.20(59.65,177.22) | 4.50(2.35,7.41) | 0.12(0.09,0.15) | 0.59(0.55,0.64) |
| Bolivia (Plurinational State of) | 225.97(129.23,325.50) | 10.45(5.90,15.06) | 287.63(164.55,439.54) | 14.20(8.03,21.53) | 0.92(0.81,1.02) | 1.19(1.08,1.31) |
| Bosnia and Herzegovina | 76.17(40.26,119.17) | 2.60(1.36,4.15) | 63.23(31.66,101.69) | 2.53(1.20,4.25) | -0.74(-1.02,-0.45) | -0.20(-0.53,0.15) |
| Botswana | 140.52(75.59,223.04) | 5.87(3.14,9.36) | 161.50(91.60,255.20) | 7.21(3.91,11.48) | 0.53(0.33,0.73) | 0.84(0.61,1.06) |
| Brazil | 133.35(83.70,181.15) | 5.14(3.30,7.03) | 123.64(76.83,167.39) | 5.13(3.20,7.01) | -0.37(-0.54,-0.21) | -0.02(-0.19,0.14) |
| Brunei Darussalam | 183.55(97.53,298.11) | 9.29(4.85,15.42) | 162.41(88.27,255.51) | 8.50(4.60,13.59) | -0.01(-0.20,0.18) | 0.36(0.08,0.64) |
| Bulgaria | 62.06(33.86,92.07) | 2.01(1.10,3.04) | 112.59(58.71,174.63) | 4.37(2.27,6.84) | 2.49(2.13,2.86) | 3.32(2.80,3.85) |
| Burkina Faso | 200.88(109.18,299.51) | 9.57(5.22,14.21) | 227.32(129.83,345.29) | 11.17(6.27,16.68) | 0.46(0.38,0.54) | 0.56(0.48,0.63) |
| Burundi | 169.49(88.81,276.71) | 7.64(4.05,12.51) | 143.15(71.27,238.38) | 6.93(3.44,11.90) | -0.98(-1.18,-0.79) | -0.74(-0.92,-0.56) |
| Cabo Verde | 92.54(51.57,132.88) | 3.75(2.12,5.46) | 128.90(65.67,202.66) | 6.32(3.04,10.00) | 0.74(0.48,1.00) | 1.33(0.98,1.68) |
| Cambodia | 141.22(76.22,212.93) | 5.56(3.07,8.38) | 141.49(75.56,224.21) | 6.17(3.25,9.55) | -0.11(-0.26,0.05) | 0.27(0.10,0.45) |
| Cameroon | 229.19(121.93,352.03) | 10.84(5.67,16.54) | 236.48(126.49,387.63) | 10.93(5.84,17.97) | -0.79(-1.31,-0.27) | -0.83(-1.30,-0.36) |
| Canada | 33.42(19.78,48.00) | 1.64(0.96,2.38) | 40.34(23.71,58.40) | 2.05(1.15,3.02) | 0.97(0.77,1.17) | 1.08(0.85,1.30) |
| Central African Republic | 278.31(155.23,416.44) | 11.45(6.58,17.36) | 276.94(146.25,444.43) | 11.61(5.92,18.72) | -0.03(-0.08,0.03) | 0.06(0.01,0.12) |
| Chad | 149.92(82.68,227.96) | 6.77(3.75,10.59) | 175.42(95.19,280.20) | 7.97(4.32,12.73) | 0.36(0.17,0.54) | 0.38(0.20,0.56) |
| Chile | 88.74(51.73,131.56) | 4.02(2.34,5.91) | 87.07(49.80,131.20) | 4.67(2.66,7.16) | 0.30(-0.11,0.72) | 0.93(0.47,1.40) |
| China | 72.02(40.77,107.15) | 3.02(1.74,4.53) | 47.98(25.31,73.67) | 2.06(1.05,3.21) | -1.31(-1.41,-1.22) | -1.31(-1.39,-1.22) |
| Colombia | 110.36(61.84,160.64) | 4.58(2.59,6.73) | 92.54(48.91,141.17) | 3.91(2.08,5.99) | -0.33(-0.50,-0.16) | -0.17(-0.36,0.02) |
| Comoros | 171.27(89.40,270.11) | 7.88(4.13,12.51) | 187.36(95.12,312.24) | 9.12(4.68,15.21) | 0.14(0.04,0.24) | 0.35(0.28,0.42) |
| Congo | 298.82(154.67,463.00) | 13.00(6.74,19.51) | 279.24(143.20,446.87) | 12.83(6.30,20.77) | -0.43(-0.56,-0.29) | -0.22(-0.34,-0.09) |
| Cook Islands | 54.90(30.16,83.96) | 2.14(1.11,3.35) | 72.28(37.56,114.68) | 3.03(1.50,4.84) | 0.95(0.86,1.03) | 1.14(1.02,1.25) |
| Costa Rica | 106.40(61.04,154.76) | 3.82(2.21,5.57) | 162.78(88.13,246.94) | 6.57(3.58,10.08) | 1.46(1.11,1.82) | 1.89(1.45,2.34) |
| Croatia | 65.37(33.87,101.59) | 2.49(1.28,3.90) | 70.34(33.96,111.88) | 3.31(1.60,5.42) | 0.31(0.09,0.54) | 1.13(0.78,1.47) |
| Cuba | 60.09(37.85,81.77) | 2.10(1.34,2.84) | 77.36(47.84,108.83) | 3.21(2.00,4.49) | 1.08(0.90,1.27) | 1.80(1.55,2.05) |
| Cyprus | 111.64(59.82,170.53) | 7.54(4.02,11.82) | 68.25(38.03,100.54) | 4.10(2.24,6.19) | -1.67(-1.92,-1.41) | -2.09(-2.42,-1.77) |
| Czechia | 69.27(36.31,104.71) | 2.25(1.16,3.49) | 46.87(24.85,72.32) | 1.74(0.90,2.78) | -1.08(-1.23,-0.93) | -0.66(-0.88,-0.45) |
| Côte d'Ivoire | 180.12(98.08,273.01) | 8.15(4.44,12.12) | 190.73(103.55,295.94) | 8.68(4.73,13.54) | 0.09(-0.05,0.22) | 0.11(-0.04,0.26) |
| Democratic People's Republic of Korea | 65.70(36.18,102.11) | 2.54(1.33,4.20) | 63.60(35.31,98.49) | 2.40(1.30,3.85) | -0.14(-0.28,0.00) | -0.09(-0.24,0.06) |
| Democratic Republic of the Congo | 231.25(133.99,349.92) | 10.04(5.73,15.39) | 233.94(131.31,363.45) | 10.33(5.68,16.14) | -0.16(-0.24,-0.08) | -0.08(-0.16,0.00) |
| Denmark | 39.66(23.34,57.34) | 1.17(0.68,1.73) | 52.99(30.63,79.47) | 2.65(1.53,3.97) | 0.79(0.65,0.93) | 2.69(2.51,2.86) |
| Djibouti | 149.31(78.14,245.35) | 6.98(3.71,11.24) | 209.02(110.44,355.05) | 10.38(5.41,17.73) | 1.04(0.89,1.20) | 1.26(1.12,1.39) |
| Dominica | 127.79(75.91,186.07) | 5.65(3.34,8.41) | 186.49(110.81,277.17) | 8.10(4.73,12.29) | 1.48(1.36,1.60) | 1.37(1.29,1.45) |
| Dominican Republic | 96.99(59.14,136.93) | 4.07(2.44,5.89) | 118.64(65.06,182.26) | 4.77(2.57,7.43) | 1.24(1.05,1.42) | 1.21(0.93,1.48) |
| Ecuador | 121.90(75.53,170.09) | 5.62(3.49,7.98) | 206.00(115.34,332.32) | 9.87(5.67,15.12) | 1.68(0.72,2.65) | 1.93(0.90,2.97) |
| Egypt | 119.30(62.55,199.43) | 6.27(3.17,10.58) | 101.03(52.11,171.62) | 5.07(2.53,8.51) | -0.34(-0.53,-0.14) | -0.43(-0.64,-0.22) |
| El Salvador | 154.55(86.03,237.51) | 5.90(3.27,9.68) | 377.06(198.88,612.51) | 15.38(8.21,25.06) | 2.96(2.51,3.42) | 3.11(2.60,3.62) |
| Equatorial Guinea | 248.99(141.92,377.86) | 10.45(6.01,15.80) | 265.22(131.89,442.98) | 12.33(5.47,20.98) | 0.27(-0.21,0.74) | 0.67(0.20,1.14) |
| Eritrea | 151.63(77.58,256.26) | 6.53(3.41,11.04) | 165.93(80.78,295.05) | 7.95(4.02,14.26) | 0.31(0.27,0.36) | 0.67(0.60,0.73) |
| Estonia | 52.84(29.51,77.28) | 1.15(0.64,1.71) | 85.96(49.53,124.01) | 3.54(2.12,5.02) | 1.12(0.65,1.59) | 3.13(2.41,3.85) |
| Eswatini | 200.76(110.36,302.31) | 8.72(4.88,13.35) | 291.23(152.69,483.05) | 12.03(6.51,19.31) | 1.51(0.83,2.20) | 1.43(0.77,2.09) |
| Ethiopia | 307.29(176.33,452.28) | 14.47(8.39,21.17) | 194.25(112.42,302.35) | 9.89(5.65,15.23) | -1.98(-2.22,-1.74) | -1.71(-1.94,-1.48) |
| Fiji | 99.40(51.18,159.91) | 3.82(1.85,6.47) | 171.55(81.96,282.98) | 7.67(3.61,12.48) | 1.42(1.13,1.70) | 1.77(1.37,2.17) |
| Finland | 31.57(17.51,45.56) | 0.79(0.44,1.15) | 30.73(16.81,46.20) | 1.06(0.52,1.60) | 0.35(0.18,0.53) | 1.51(1.22,1.79) |
| France | 40.07(23.40,56.85) | 1.94(1.16,2.74) | 39.80(23.78,57.81) | 1.87(1.11,2.70) | 0.13(0.05,0.22) | 0.40(0.19,0.61) |
| Gabon | 214.97(117.88,323.46) | 9.62(5.16,14.31) | 296.43(141.96,478.87) | 14.23(6.28,22.90) | 0.97(0.76,1.18) | 1.20(0.98,1.41) |
| Gambia | 191.44(106.16,281.93) | 8.86(5.00,12.94) | 245.13(133.74,375.50) | 11.49(6.19,17.72) | 0.64(0.50,0.78) | 0.71(0.60,0.81) |
| Georgia | 50.05(26.20,76.48) | 0.59(0.27,0.99) | 89.83(45.85,136.88) | 2.39(1.09,3.81) | 2.03(1.62,2.44) | 4.67(3.91,5.43) |
| Germany | 54.01(31.14,78.09) | 1.93(1.12,2.81) | 60.89(33.92,89.40) | 3.20(1.70,4.82) | 0.92(0.69,1.15) | 2.90(2.38,3.41) |
| Ghana | 133.04(72.82,209.61) | 6.17(3.45,9.58) | 186.47(98.99,296.10) | 9.12(4.87,14.40) | 1.28(1.18,1.38) | 1.47(1.36,1.57) |
| Greece | 48.65(22.24,82.11) | 2.81(1.26,4.77) | 53.37(26.29,86.14) | 2.87(1.44,4.62) | 0.11(-0.62,0.85) | -0.42(-1.55,0.71) |
| Greenland | 64.33(37.92,91.57) | 3.02(1.81,4.38) | 62.99(35.33,94.39) | 3.01(1.62,4.54) | 0.32(0.18,0.45) | 0.44(0.27,0.61) |
| Grenada | 214.35(131.51,297.87) | 8.29(5.01,11.49) | 310.64(193.44,436.61) | 13.18(7.77,18.70) | 1.65(1.45,1.86) | 1.86(1.57,2.15) |
| Guam | 104.18(61.15,154.64) | 4.82(2.82,7.28) | 111.35(65.35,167.78) | 3.85(2.26,5.75) | 0.91(0.65,1.17) | 0.19(-0.19,0.57) |
| Guatemala | 161.24(95.27,229.81) | 7.29(4.29,10.32) | 268.12(144.75,414.71) | 10.76(5.91,16.63) | 2.59(2.10,3.08) | 2.14(1.68,2.61) |
| Guinea | 129.10(69.91,200.05) | 5.85(3.11,9.18) | 153.77(81.03,247.96) | 6.88(3.56,10.99) | 0.67(0.57,0.77) | 0.66(0.55,0.77) |
| Guinea-Bissau | 268.74(146.27,408.96) | 11.59(6.36,17.43) | 248.33(135.37,389.44) | 11.05(6.08,17.27) | -0.30(-0.34,-0.26) | -0.19(-0.23,-0.15) |
| Guyana | 203.72(127.92,279.39) | 8.06(4.95,11.18) | 341.02(204.36,508.19) | 13.45(8.04,20.35) | 2.65(2.29,3.02) | 2.69(2.30,3.08) |
| Haiti | 186.40(102.70,325.11) | 7.10(3.80,13.64) | 207.63(99.28,417.20) | 8.03(3.75,17.16) | 0.57(0.49,0.65) | 0.60(0.53,0.68) |
| Honduras | 84.46(46.90,130.13) | 2.73(1.51,4.32) | 137.71(72.70,215.72) | 5.44(2.82,8.71) | 1.81(1.62,1.99) | 2.48(2.19,2.78) |
| Hungary | 62.04(33.94,92.06) | 1.90(1.00,2.83) | 65.23(35.16,102.01) | 2.88(1.50,4.56) | 0.75(0.50,1.01) | 2.36(2.01,2.72) |
| Iceland | 29.79(17.42,42.21) | 0.98(0.61,1.39) | 33.69(19.19,49.28) | 1.41(0.78,2.05) | 0.55(0.44,0.66) | 1.67(1.48,1.85) |
| India | 90.10(49.23,134.23) | 2.92(1.63,4.21) | 101.71(55.62,155.58) | 3.50(1.88,5.38) | 0.35(0.31,0.40) | 0.52(0.36,0.68) |
| Indonesia | 142.78(79.98,213.71) | 5.14(2.97,7.70) | 157.54(90.00,246.44) | 6.26(3.59,9.68) | 0.34(0.28,0.40) | 0.65(0.55,0.76) |
| Iran (Islamic Republic of) | 54.47(30.14,82.13) | 2.51(1.32,3.93) | 36.15(19.48,55.32) | 1.80(0.92,2.73) | -1.44(-1.76,-1.12) | -1.25(-1.56,-0.93) |
| Iraq | 121.94(64.67,189.74) | 5.20(2.77,8.06) | 132.88(68.80,216.68) | 6.46(3.32,10.38) | -0.30(-0.47,-0.12) | 0.07(-0.13,0.27) |
| Ireland | 58.62(33.19,84.85) | 2.00(1.17,2.86) | 48.01(28.02,70.81) | 1.73(0.98,2.56) | -0.44(-0.54,-0.35) | 0.19(-0.04,0.41) |
| Israel | 52.54(27.00,85.40) | 2.94(1.52,4.90) | 40.96(20.30,70.17) | 2.17(1.06,3.68) | -0.21(-0.57,0.16) | -0.16(-0.66,0.34) |
| Italy | 40.52(21.54,61.31) | 1.70(0.92,2.68) | 35.08(18.94,52.16) | 1.74(0.92,2.62) | -0.52(-0.66,-0.37) | 0.06(-0.15,0.26) |
| Jamaica | 118.59(70.73,163.27) | 5.44(3.17,7.54) | 162.80(94.15,242.52) | 6.50(3.72,10.08) | 0.71(0.12,1.30) | 0.13(-0.51,0.77) |
| Japan | 74.39(41.76,109.81) | 3.26(1.80,4.95) | 49.92(27.22,74.37) | 2.14(1.17,3.33) | -1.40(-1.51,-1.30) | -1.55(-1.68,-1.41) |
| Jordan | 111.37(59.98,169.02) | 5.27(2.72,8.05) | 111.91(64.26,164.91) | 5.68(3.16,8.48) | -0.22(-0.52,0.09) | 0.05(-0.25,0.34) |
| Kazakhstan | 76.76(42.23,115.60) | 1.13(0.60,1.75) | 75.17(39.13,117.90) | 2.14(1.03,3.59) | -0.66(-0.98,-0.33) | 1.38(0.95,1.81) |
| Kenya | 117.62(62.92,190.36) | 5.61(2.94,9.08) | 167.02(92.17,257.91) | 8.24(4.50,12.73) | 1.40(1.30,1.50) | 1.51(1.41,1.61) |
| Kiribati | 84.53(45.39,132.26) | 3.24(1.69,5.21) | 110.62(52.87,190.29) | 4.61(2.04,8.14) | 0.74(0.56,0.92) | 0.99(0.75,1.22) |
| Kuwait | 88.71(49.27,129.86) | 4.21(2.25,6.25) | 47.67(24.47,76.08) | 2.36(1.20,3.84) | -1.86(-2.05,-1.67) | -1.80(-2.03,-1.57) |
| Kyrgyzstan | 57.11(32.00,87.06) | 0.65(0.31,1.06) | 62.82(34.65,95.21) | 1.35(0.66,2.20) | -0.58(-1.09,-0.08) | 0.74(-0.16,1.65) |
| Lao People's Democratic Republic | 290.81(156.03,437.40) | 11.70(6.31,17.74) | 205.45(104.77,345.32) | 9.05(4.55,14.79) | -1.38(-1.47,-1.28) | -1.06(-1.15,-0.97) |
| Latvia | 37.84(21.33,55.30) | 0.53(0.29,0.80) | 60.34(33.86,90.36) | 1.71(0.91,2.61) | 1.34(1.14,1.54) | 3.81(3.31,4.32) |
| Lebanon | 36.88(17.57,66.26) | 1.78(0.87,3.18) | 48.14(26.14,78.36) | 2.47(1.28,4.05) | 1.39(1.11,1.67) | 1.60(1.33,1.87) |
| Lesotho | 111.33(64.53,168.15) | 4.69(2.57,7.24) | 260.97(137.15,419.05) | 11.06(5.88,17.60) | 3.62(3.11,4.12) | 3.74(3.23,4.26) |
| Liberia | 229.00(127.80,353.63) | 10.72(5.91,15.99) | 252.55(136.54,397.17) | 11.80(6.15,18.91) | 0.54(0.24,0.84) | 0.53(0.25,0.81) |
| Libya | 79.96(44.12,125.60) | 3.66(1.97,5.89) | 150.57(79.34,240.47) | 7.16(3.60,11.48) | 2.52(2.37,2.67) | 2.80(2.61,2.98) |
| Lithuania | 37.77(21.20,55.95) | 0.48(0.26,0.73) | 54.19(30.14,82.91) | 1.32(0.72,2.03) | 0.69(0.46,0.92) | 2.05(1.51,2.59) |
| Luxembourg | 61.44(36.95,88.51) | 2.57(1.48,3.71) | 53.91(31.10,78.94) | 2.49(1.33,3.80) | -0.23(-0.41,-0.06) | 0.25(-0.03,0.53) |
| Madagascar | 126.70(64.78,207.83) | 5.74(2.97,9.40) | 135.54(71.07,219.37) | 6.30(3.23,10.20) | 0.27(0.19,0.34) | 0.34(0.26,0.43) |
| Malawi | 198.38(100.01,313.06) | 9.07(4.68,14.41) | 227.89(120.60,362.23) | 10.71(5.70,17.20) | 0.23(-0.03,0.50) | 0.36(0.13,0.59) |
| Malaysia | 176.27(104.87,255.72) | 6.94(4.04,9.95) | 179.45(101.85,270.30) | 7.72(4.51,11.68) | -0.07(-0.21,0.06) | 0.17(-0.01,0.35) |
| Maldives | 300.57(169.97,451.03) | 13.00(7.17,20.48) | 139.05(73.05,218.55) | 6.93(3.63,10.87) | -2.89(-3.12,-2.65) | -2.39(-2.60,-2.17) |
| Mali | 196.88(108.30,296.39) | 9.29(5.07,13.95) | 186.41(101.65,283.69) | 9.03(4.84,13.63) | -0.08(-0.23,0.06) | 0.03(-0.11,0.16) |
| Malta | 65.72(37.35,95.33) | 2.80(1.58,4.04) | 54.61(29.34,83.28) | 2.32(1.22,3.54) | -0.47(-0.65,-0.30) | -0.39(-0.65,-0.12) |
| Marshall Islands | 86.49(39.63,161.25) | 3.39(1.45,6.47) | 153.53(43.56,394.75) | 6.53(1.49,18.36) | 1.94(1.79,2.08) | 2.20(2.04,2.36) |
| Mauritania | 242.14(135.96,365.10) | 11.35(6.36,16.98) | 224.16(120.13,356.18) | 11.23(5.83,18.14) | -0.56(-0.73,-0.38) | -0.35(-0.54,-0.16) |
| Mauritius | 277.92(161.07,389.87) | 11.81(6.82,16.86) | 455.36(266.72,656.57) | 19.90(11.54,28.83) | 1.81(1.40,2.22) | 1.87(1.47,2.28) |
| Mexico | 170.36(104.15,240.18) | 7.66(4.57,11.15) | 273.20(158.47,401.65) | 10.76(6.09,15.76) | 2.14(1.50,2.77) | 1.81(1.17,2.44) |
| Micronesia (Federated States of) | 103.56(50.50,179.70) | 4.25(1.96,8.04) | 152.45(75.79,258.51) | 6.56(3.00,11.38) | 1.34(1.12,1.55) | 1.48(1.21,1.75) |
| Monaco | 32.54(16.98,51.95) | 1.15(0.56,1.86) | 38.55(18.20,64.17) | 1.63(0.74,2.68) | 0.70(0.54,0.86) | 1.39(1.08,1.70) |
| Mongolia | 159.11(86.41,236.63) | 4.23(2.08,6.39) | 128.41(69.95,189.16) | 3.53(1.86,5.46) | -1.05(-1.20,-0.90) | -1.07(-1.25,-0.89) |
| Montenegro | 77.07(42.00,120.77) | 3.19(1.69,5.11) | 79.92(37.19,139.55) | 4.01(1.83,7.33) | -0.05(-0.29,0.20) | 0.69(0.26,1.11) |
| Morocco | 99.31(47.12,176.04) | 4.42(2.03,9.09) | 91.30(47.97,139.52) | 4.54(2.34,7.02) | -0.24(-0.59,0.10) | 0.14(-0.22,0.51) |
| Mozambique | 137.51(67.10,223.87) | 6.34(3.20,10.57) | 189.73(93.56,317.58) | 8.89(4.54,14.82) | 1.43(1.25,1.60) | 1.49(1.33,1.64) |
| Myanmar | 186.39(105.80,292.33) | 7.19(4.22,11.09) | 147.35(80.67,230.57) | 6.28(3.47,9.95) | -1.02(-1.12,-0.93) | -0.72(-0.82,-0.62) |
| Namibia | 139.33(76.45,217.53) | 5.74(3.13,9.05) | 153.55(82.86,233.91) | 6.65(3.52,10.21) | 0.04(-0.30,0.37) | 0.25(-0.08,0.58) |
| Nauru | 112.75(56.19,212.07) | 4.55(2.13,9.28) | 179.27(88.99,327.60) | 7.50(3.59,14.44) | 1.35(1.20,1.49) | 1.41(1.28,1.55) |
| Nepal | 99.61(55.18,152.41) | 3.01(1.67,4.67) | 113.02(64.68,173.28) | 3.91(2.17,6.25) | 0.41(0.18,0.65) | 1.11(0.78,1.45) |
| Netherlands | 36.26(20.79,51.82) | 1.38(0.79,2.05) | 46.95(27.16,67.76) | 2.38(1.38,3.45) | 0.95(0.70,1.20) | 2.07(1.65,2.49) |
| New Zealand | 40.11(23.48,57.77) | 1.58(0.93,2.25) | 49.81(29.25,71.96) | 2.20(1.25,3.17) | 0.92(0.55,1.29) | 1.32(0.84,1.79) |
| Nicaragua | 196.12(107.09,299.76) | 7.22(3.84,11.19) | 379.74(200.66,595.50) | 14.24(7.61,22.21) | 2.66(2.21,3.10) | 2.79(2.30,3.27) |
| Niger | 167.98(94.90,249.75) | 7.49(4.27,11.26) | 145.37(80.57,229.64) | 6.81(3.71,10.88) | -0.53(-0.64,-0.41) | -0.34(-0.44,-0.25) |
| Nigeria | 138.37(76.67,204.80) | 6.26(3.45,9.37) | 149.47(78.49,228.58) | 6.88(3.61,10.46) | 0.28(0.22,0.35) | 0.30(0.24,0.36) |
| Niue | 96.24(51.86,164.20) | 3.89(2.02,6.73) | 174.81(71.38,338.94) | 7.93(3.05,15.51) | 1.87(1.77,1.98) | 2.27(2.13,2.41) |
| North Macedonia | 68.95(36.34,109.60) | 2.32(1.16,3.78) | 68.89(32.47,116.89) | 2.85(1.30,5.06) | -0.06(-0.28,0.16) | 0.57(0.18,0.95) |
| Northern Mariana Islands | 105.06(46.56,165.04) | 4.61(1.90,7.46) | 125.78(60.16,204.07) | 5.65(2.52,9.31) | 0.69(0.49,0.90) | 0.70(0.48,0.92) |
| Norway | 36.99(21.71,53.99) | 0.98(0.58,1.39) | 42.33(24.67,60.80) | 1.75(1.01,2.50) | 0.59(0.45,0.74) | 2.28(1.93,2.62) |
| Oman | 68.75(36.72,108.91) | 3.05(1.63,4.94) | 100.82(57.68,154.57) | 4.96(2.77,7.72) | 1.92(1.58,2.27) | 2.34(1.86,2.82) |
| Pakistan | 117.84(65.48,180.30) | 4.45(2.42,6.60) | 166.44(90.55,251.59) | 6.36(3.37,9.71) | 0.97(0.80,1.15) | 1.01(0.78,1.23) |
| Palau | 88.29(45.54,133.04) | 3.59(1.75,5.59) | 142.12(68.63,228.82) | 6.45(3.03,10.70) | 1.70(1.56,1.85) | 2.13(1.95,2.30) |
| Palestine | 179.16(93.76,271.42) | 9.21(4.82,14.24) | 124.87(69.58,188.68) | 6.83(3.63,10.24) | -1.49(-1.60,-1.38) | -1.23(-1.38,-1.09) |
| Panama | 95.64(54.78,137.37) | 3.39(1.96,4.97) | 177.77(100.32,275.47) | 7.41(4.09,11.74) | 2.25(1.84,2.66) | 2.83(2.31,3.36) |
| Papua New Guinea | 35.73(18.64,55.37) | 1.14(0.55,1.86) | 36.78(21.44,56.75) | 1.21(0.66,2.00) | 0.04(0.00,0.08) | 0.13(0.07,0.20) |
| Paraguay | 114.64(69.25,160.11) | 4.78(2.88,6.71) | 155.49(91.30,234.33) | 6.74(3.86,10.31) | 1.02(0.85,1.18) | 1.30(1.14,1.46) |
| Peru | 147.79(90.96,208.79) | 7.05(4.26,9.97) | 159.12(87.49,239.05) | 7.87(4.26,11.79) | 0.03(-0.19,0.25) | 0.13(-0.09,0.36) |
| Philippines | 163.59(92.36,243.93) | 7.51(4.14,11.17) | 209.55(108.16,319.57) | 8.82(4.57,13.55) | 1.11(0.99,1.24) | 0.91(0.78,1.03) |
| Poland | 77.28(42.21,116.15) | 2.90(1.58,4.36) | 40.89(21.06,62.29) | 1.42(0.75,2.21) | -1.91(-2.35,-1.46) | -2.25(-2.95,-1.55) |
| Portugal | 50.45(28.49,73.99) | 2.25(1.27,3.27) | 44.04(23.93,65.84) | 2.27(1.24,3.47) | -0.14(-0.57,0.29) | 0.16(-0.35,0.66) |
| Puerto Rico | 178.37(109.02,246.47) | 8.09(4.97,11.22) | 161.26(96.21,230.13) | 7.08(4.15,10.24) | 0.18(-0.20,0.55) | 0.16(-0.31,0.63) |
| Qatar | 64.44(29.75,113.13) | 3.32(1.52,6.03) | 66.01(30.68,108.47) | 3.55(1.68,5.83) | -0.45(-0.89,-0.02) | -0.47(-1.01,0.06) |
| Republic of Korea | 77.61(42.60,120.54) | 4.27(2.36,6.61) | 40.63(22.17,61.13) | 2.40(1.31,3.61) | -2.07(-2.22,-1.91) | -1.92(-2.15,-1.69) |
| Republic of Moldova | 29.64(16.33,45.13) | 0.29(0.15,0.44) | 43.76(23.78,64.73) | 0.64(0.34,0.98) | 0.96(0.77,1.14) | 1.96(1.52,2.41) |
| Romania | 81.01(42.42,119.39) | 2.71(1.37,4.18) | 66.79(34.86,103.85) | 2.14(1.06,3.50) | -0.23(-0.64,0.18) | -0.48(-1.06,0.11) |
| Russian Federation | 49.27(27.86,71.97) | 1.00(0.57,1.49) | 41.96(23.35,61.82) | 1.29(0.70,1.95) | -1.14(-1.42,-0.86) | 0.26(-0.29,0.82) |
| Rwanda | 170.75(86.71,280.09) | 7.77(3.89,12.75) | 121.38(59.53,205.76) | 6.18(2.94,10.60) | -2.06(-2.45,-1.66) | -1.60(-1.97,-1.23) |
| Saint Kitts and Nevis | 295.90(190.18,390.45) | 12.31(7.81,16.56) | 331.11(207.58,466.65) | 15.30(9.36,21.83) | 1.13(0.77,1.49) | 1.63(1.28,1.97) |
| Saint Lucia | 204.41(126.96,279.76) | 9.05(5.47,12.53) | 230.43(142.82,321.56) | 9.93(6.01,14.20) | 0.88(0.61,1.15) | 0.65(0.34,0.96) |
| Saint Vincent and the Grenadines | 167.58(107.01,225.16) | 6.97(4.27,9.47) | 214.86(133.96,300.13) | 9.20(5.57,12.90) | 1.24(0.92,1.57) | 1.42(1.05,1.79) |
| Samoa | 86.51(44.87,139.12) | 3.49(1.71,5.68) | 128.75(67.12,207.22) | 5.48(2.76,8.62) | 1.31(1.24,1.37) | 1.47(1.40,1.54) |
| San Marino | 34.00(19.76,50.44) | 1.21(0.68,1.81) | 28.40(15.88,43.76) | 0.80(0.38,1.29) | -0.01(-0.23,0.20) | -0.06(-0.53,0.42) |
| Sao Tome and Principe | 239.75(129.76,355.77) | 11.67(6.39,17.45) | 286.11(148.15,462.27) | 14.34(7.17,23.15) | 0.50(0.40,0.59) | 0.72(0.68,0.77) |
| Saudi Arabia | 139.79(75.37,212.56) | 6.38(3.42,10.23) | 275.97(159.28,426.24) | 12.91(7.56,19.76) | 2.07(1.75,2.39) | 2.13(1.83,2.43) |
| Senegal | 223.05(124.52,330.80) | 10.35(5.59,15.28) | 216.71(118.33,351.27) | 10.70(5.70,16.88) | -0.14(-0.27,-0.01) | 0.02(-0.10,0.14) |
| Serbia | 102.08(54.11,158.31) | 4.73(2.45,7.55) | 84.13(43.87,133.67) | 4.06(2.12,6.61) | -0.83(-0.96,-0.70) | -0.61(-0.77,-0.44) |
| Seychelles | 196.57(114.43,285.45) | 7.90(4.52,11.48) | 247.09(134.24,375.95) | 11.39(6.18,17.49) | 0.83(0.63,1.04) | 1.29(1.04,1.55) |
| Sierra Leone | 167.06(91.61,251.86) | 7.78(4.28,11.69) | 162.09(86.63,252.07) | 7.41(3.88,11.40) | -0.09(-0.15,-0.02) | -0.19(-0.26,-0.12) |
| Singapore | 66.10(35.59,101.38) | 2.87(1.54,4.39) | 37.40(20.18,58.43) | 1.73(0.91,2.76) | -1.13(-1.64,-0.62) | -0.69(-1.34,-0.03) |
| Slovakia | 90.37(48.79,137.68) | 3.17(1.74,4.87) | 67.36(37.12,103.14) | 2.54(1.39,4.02) | -0.93(-1.00,-0.86) | -0.62(-0.71,-0.53) |
| Slovenia | 55.13(27.85,84.19) | 1.72(0.83,2.72) | 46.38(24.35,71.97) | 1.70(0.85,2.78) | -0.40(-0.55,-0.25) | 0.75(0.42,1.08) |
| Solomon Islands | 75.95(32.11,133.25) | 2.85(1.03,5.06) | 65.68(37.60,102.68) | 2.38(1.26,3.79) | -0.65(-0.84,-0.46) | -0.80(-1.03,-0.57) |
| Somalia | 222.99(119.02,374.02) | 10.07(5.32,16.61) | 248.27(127.87,426.47) | 11.21(5.79,19.00) | 0.46(0.35,0.56) | 0.52(0.40,0.63) |
| South Africa | 109.24(59.30,165.54) | 4.04(2.16,6.07) | 187.67(105.40,270.94) | 8.11(4.50,11.77) | 1.46(1.03,1.90) | 1.90(1.38,2.42) |
| South Sudan | 179.82(94.64,288.57) | 8.46(4.51,13.80) | 254.72(131.70,427.17) | 12.18(6.17,20.18) | 1.01(0.78,1.23) | 1.08(0.90,1.26) |
| Spain | 43.82(24.25,66.41) | 2.08(1.20,3.07) | 32.85(18.51,48.31) | 1.57(0.88,2.37) | -0.55(-0.71,-0.39) | -0.57(-0.71,-0.44) |
| Sri Lanka | 176.86(98.34,256.83) | 8.13(4.44,11.96) | 130.73(67.47,216.57) | 5.65(2.92,9.55) | -0.98(-1.16,-0.81) | -1.12(-1.29,-0.95) |
| Sudan | 106.19(56.67,184.10) | 4.52(2.27,8.59) | 131.34(70.34,210.22) | 6.01(3.28,9.70) | 0.72(0.59,0.85) | 0.99(0.84,1.14) |
| Suriname | 173.41(107.31,242.48) | 6.65(4.06,9.50) | 232.15(134.97,343.97) | 9.12(5.18,13.88) | 1.22(1.01,1.42) | 1.39(1.18,1.61) |
| Sweden | 31.14(17.94,45.32) | 0.93(0.55,1.37) | 41.17(23.24,61.22) | 2.04(1.12,3.05) | 1.27(1.13,1.41) | 2.93(2.75,3.11) |
| Switzerland | 37.81(22.06,54.33) | 1.48(0.86,2.13) | 36.17(20.76,53.63) | 1.77(0.99,2.69) | 0.33(0.13,0.53) | 1.57(1.19,1.96) |
| Syrian Arab Republic | 108.28(64.24,170.34) | 5.18(3.01,8.07) | 145.45(78.54,239.01) | 7.36(3.75,12.09) | 0.45(0.15,0.75) | 0.65(0.38,0.93) |
| Taiwan (Province of China) | 98.99(55.79,147.25) | 5.10(2.89,7.41) | 65.57(32.93,99.88) | 3.31(1.68,5.09) | -0.79(-1.09,-0.49) | -1.07(-1.45,-0.69) |
| Tajikistan | 32.35(16.06,52.78) | 0.23(0.08,0.42) | 32.00(16.15,50.74) | 0.29(0.11,0.56) | -0.43(-0.66,-0.20) | 0.08(-0.51,0.67) |
| Thailand | 132.55(74.98,204.95) | 5.81(3.25,9.06) | 138.04(69.67,219.51) | 6.20(3.06,9.89) | -0.39(-0.67,-0.12) | -0.27(-0.56,0.01) |
| Timor-Leste | 181.56(99.33,278.89) | 7.59(4.09,11.56) | 203.06(106.76,329.35) | 8.83(4.61,14.23) | 0.53(0.30,0.77) | 0.69(0.46,0.92) |
| Togo | 177.10(97.53,257.14) | 8.20(4.51,11.83) | 200.06(111.61,304.24) | 9.45(5.21,14.79) | 0.28(0.16,0.40) | 0.34(0.23,0.45) |
| Tokelau | 68.94(35.86,123.81) | 2.71(1.32,5.28) | 102.02(54.35,168.37) | 4.43(2.18,7.74) | 1.35(1.27,1.42) | 1.72(1.62,1.82) |
| Tonga | 47.29(24.60,75.60) | 1.51(0.72,2.65) | 68.29(34.75,114.11) | 2.55(1.25,4.29) | 1.07(0.96,1.18) | 1.60(1.42,1.78) |
| Trinidad and Tobago | 163.78(101.81,218.76) | 6.84(4.20,9.24) | 270.12(167.42,398.50) | 10.78(6.74,16.22) | 2.10(1.76,2.44) | 2.14(1.75,2.53) |
| Tunisia | 72.54(38.62,116.68) | 3.33(1.70,5.53) | 68.72(36.71,110.03) | 3.50(1.84,5.71) | -0.41(-0.56,-0.25) | -0.04(-0.21,0.14) |
| Turkey | 54.32(29.56,86.78) | 2.58(1.39,4.19) | 36.43(18.33,60.66) | 1.79(0.91,3.16) | -1.06(-1.35,-0.76) | -0.89(-1.33,-0.45) |
| Turkmenistan | 80.86(43.50,120.92) | 1.53(0.79,2.42) | 94.82(49.48,161.69) | 2.49(1.22,4.42) | 0.07(-0.37,0.52) | 0.85(0.14,1.56) |
| Tuvalu | 84.66(44.93,144.79) | 3.24(1.62,5.99) | 117.21(62.70,196.10) | 4.86(2.36,8.20) | 1.12(1.08,1.15) | 1.41(1.36,1.45) |
| Uganda | 126.90(61.59,214.60) | 5.97(2.89,10.14) | 148.33(72.61,253.13) | 7.24(3.58,12.62) | 0.14(-0.11,0.39) | 0.33(0.12,0.55) |
| Ukraine | 16.10(8.43,25.44) | 0.02(0.01,0.03) | 25.34(13.82,39.92) | 0.32(0.16,0.54) | 1.78(1.51,2.05) | 12.63(10.57,14.73) |
| United Arab Emirates | 49.33(24.14,84.70) | 1.98(0.96,3.53) | 113.04(60.00,173.75) | 5.67(2.74,8.96) | 4.90(4.13,5.67) | 6.30(5.29,7.31) |
| United Kingdom | 42.03(24.16,60.72) | 1.08(0.61,1.57) | 40.14(22.96,58.70) | 1.20(0.66,1.80) | 0.09(-0.07,0.25) | 1.13(0.73,1.53) |
| United Republic of Tanzania | 183.69(95.23,288.07) | 8.35(4.37,13.33) | 203.95(106.69,313.97) | 9.50(4.91,15.09) | 0.34(0.31,0.37) | 0.38(0.34,0.42) |
| United States of America | 60.66(36.88,85.92) | 2.29(1.45,3.15) | 135.45(83.00,187.12) | 5.85(3.54,7.98) | 2.97(2.76,3.18) | 3.45(3.22,3.67) |
| United States Virgin Islands | 120.26(74.55,163.77) | 4.71(2.89,6.67) | 116.84(66.71,170.07) | 4.41(2.46,6.71) | 0.30(0.11,0.48) | 0.15(-0.10,0.40) |
| Uruguay | 72.77(42.30,105.28) | 3.54(2.05,5.02) | 75.34(41.71,110.89) | 3.88(2.19,5.74) | 0.33(0.13,0.53) | 0.59(0.34,0.84) |
| Uzbekistan | 51.57(27.76,81.58) | 0.80(0.32,1.71) | 70.17(36.23,115.21) | 1.75(0.83,3.03) | 0.35(-0.15,0.86) | 1.28(0.38,2.20) |
| Vanuatu | 90.09(42.39,165.74) | 3.55(1.57,6.96) | 114.23(59.05,206.03) | 4.52(2.21,8.19) | 0.70(0.63,0.77) | 0.70(0.62,0.79) |
| Venezuela (Bolivarian Republic of) | 113.35(63.12,165.96) | 3.97(2.26,5.80) | 223.12(119.26,357.41) | 9.29(4.85,15.06) | 1.71(1.28,2.14) | 2.36(1.88,2.85) |
| Viet Nam | 143.13(76.38,219.73) | 6.47(3.42,10.01) | 130.32(67.50,207.13) | 6.48(3.36,10.31) | -0.13(-0.29,0.03) | 0.12(0.00,0.24) |
| Yemen | 99.22(47.72,179.89) | 4.23(2.04,8.47) | 98.61(50.45,161.54) | 4.49(2.23,7.49) | -0.15(-0.29,-0.01) | 0.08(-0.07,0.23) |
| Zambia | 207.79(113.26,315.29) | 9.87(5.36,15.45) | 253.70(133.89,422.43) | 11.67(6.33,19.31) | 0.38(0.23,0.53) | 0.28(0.12,0.45) |
| Zimbabwe | 157.19(85.15,236.93) | 7.10(3.76,10.64) | 259.38(134.95,413.54) | 11.24(5.96,17.55) | 1.97(1.39,2.55) | 1.91(1.36,2.46) |

Supplementary Table 2. Age-Standardized Mortality and DALY Rates (ASMR and ASDR) for Chronic Kidney Disease (CKD) Attributable to Dietary Risk Factors in 2021

|  | Deaths | Deaths | Deaths | Deaths | Deaths | Deaths | Deaths | Deaths | DALYs (Disability-Adjusted Life Years) | DALYs (Disability-Adjusted Life Years) | DALYs (Disability-Adjusted Life Years) | DALYs (Disability-Adjusted Life Years) | DALYs (Disability-Adjusted Life Years) | DALYs (Disability-Adjusted Life Years) | DALYs (Disability-Adjusted Life Years) | DALYs (Disability-Adjusted Life Years) |
| --- | --- | --- | --- | --- | --- | --- | --- | --- | --- | --- | --- | --- | --- | --- | --- | --- |
|  | Dietary risks | Diet low in fruits | Diet low in vegetables | Diet low in whole grains | Diet high in red meat | Diet high in processed meat | Diet high in sugar-sweetened beverages | Diet high in sodium | Dietary risks | Diet low in fruits | Diet low in vegetables | Diet low in whole grains | Diet high in red meat | Diet high in processed meat | Diet high in sugar-sweetened beverages | Diet high in sodium |
| global | 3.83(2.25,5.49) | 1.53(0.79,2.31) | 1.27(0.62,2.06) | 0.27(0.07,0.50) | 0.23(0.00,0.50) | 0.25(0.06,0.44) | 0.08(0.04,0.14) | 0.84(0.09,2.39) | 93.52(54.29,134.38) | 38.68(20.15,57.77) | 30.84(14.80,50.20) | 6.46(1.59,12.52) | 5.50(0.00,12.01) | 5.95(1.53,11.00) | 2.22(1.02,3.74) | 19.81(2.51,54.57) |
| Central Europe, eastern Europe, and central Asia | 1.60(0.87,2.45) | 0.63(0.30,0.98) | 0.31(0.14,0.53) | 0.09(0.02,0.18) | 0.10(0.00,0.23) | 0.14(0.04,0.29) | 0.03(0.01,0.06) | 0.53(0.09,1.28) | 50.82(28.19,75.79) | 21.91(10.92,33.09) | 9.14(4.20,15.66) | 3.17(0.76,6.33) | 3.56(0.00,8.35) | 5.19(1.31,10.51) | 1.23(0.52,2.33) | 13.95(2.28,35.25) |
| High-income | 3.21(1.87,4.60) | 1.13(0.58,1.70) | 0.90(0.44,1.45) | 0.23(0.06,0.43) | 0.27(0.00,0.59) | 0.55(0.15,0.96) | 0.13(0.06,0.21) | 0.52(0.02,1.69) | 72.66(43.74,101.75) | 24.73(12.64,36.16) | 18.51(9.13,30.01) | 5.15(1.30,9.61) | 6.63(0.00,14.41) | 15.26(4.01,27.71) | 3.75(1.77,6.31) | 11.05(0.47,34.65) |
| Latin America and Caribbean | 7.10(4.28,10.13) | 2.23(1.21,3.27) | 3.28(1.63,5.05) | 0.45(0.11,0.81) | 0.49(0.00,1.05) | 0.22(0.06,0.39) | 0.22(0.10,0.36) | 1.49(0.07,4.42) | 172.76(104.57,243.20) | 55.15(30.04,81.09) | 78.27(39.34,123.74) | 11.42(2.84,21.43) | 13.39(0.00,29.49) | 6.16(1.63,11.35) | 6.79(3.12,11.71) | 33.21(1.66,98.54) |
| North Africa and Middle East | 4.11(2.28,6.09) | 1.91(0.95,2.96) | 1.29(0.59,2.23) | 0.49(0.12,0.95) | 0.19(0.00,0.44) | 0.12(0.03,0.23) | 0.12(0.05,0.21) | 0.33(0.00,1.88) | 87.79(49.91,130.38) | 40.92(20.80,63.09) | 25.98(12.19,44.76) | 10.96(2.76,21.13) | 4.38(0.00,9.99) | 2.96(0.73,5.54) | 3.34(1.47,5.64) | 7.13(0.00,39.56) |
| South Asia | 3.67(1.96,5.62) | 2.05(1.05,3.13) | 1.47(0.73,2.36) | 0.20(0.05,0.39) | 0.04(0.00,0.09) | 0.07(0.02,0.12) | 0.03(0.01,0.06) | 0.57(0.02,1.89) | 105.16(58.46,160.45) | 59.31(30.30,90.71) | 41.97(20.30,69.06) | 5.94(1.46,11.80) | 1.07(0.00,2.55) | 1.98(0.49,3.84) | 1.09(0.45,1.94) | 16.45(0.74,52.26) |
| Southeast Asia, east Asia, and Oceania | 3.14(1.75,4.70) | 1.14(0.58,1.78) | 0.71(0.33,1.15) | 0.25(0.06,0.47) | 0.25(0.00,0.56) | 0.07(0.02,0.13) | 0.03(0.02,0.06) | 1.14(0.20,2.73) | 74.66(42.13,111.48) | 26.94(13.86,40.96) | 16.81(7.98,27.60) | 5.68(1.40,11.08) | 5.92(0.00,12.74) | 1.81(0.44,3.30) | 0.89(0.40,1.48) | 27.10(5.43,62.61) |
| Sub-Saharan Africa | 8.75(4.86,12.97) | 4.05(2.03,6.25) | 4.24(2.03,6.81) | 0.41(0.10,0.78) | 0.15(0.00,0.36) | 0.23(0.06,0.43) | 0.05(0.02,0.08) | 1.22(0.03,4.32) | 187.27(106.65,275.11) | 87.83(44.32,132.84) | 90.48(42.80,147.65) | 9.31(2.30,17.77) | 3.75(0.00,8.65) | 5.79(1.48,11.24) | 1.25(0.54,2.20) | 23.57(0.63,83.44) |
| Andean Latin America | 9.27(5.38,13.50) | 3.03(1.58,4.60) | 4.83(2.46,7.71) | 0.63(0.16,1.16) | 0.40(0.00,0.90) | 0.15(0.04,0.28) | 0.24(0.11,0.41) | 1.90(0.04,5.86) | 191.06(112.76,273.42) | 63.65(33.78,95.28) | 98.81(49.94,155.87) | 13.99(3.55,25.69) | 9.51(0.00,21.00) | 3.87(1.04,7.11) | 6.28(2.81,10.80) | 36.32(0.91,113.36) |
| Australasia | 2.03(1.17,2.88) | 0.80(0.41,1.25) | 0.67(0.33,1.13) | 0.15(0.04,0.29) | 0.28(0.00,0.66) | 0.23(0.06,0.44) | 0.07(0.03,0.12) | 0.13(0.00,0.61) | 42.46(24.39,60.99) | 16.41(8.49,24.58) | 12.97(6.18,21.90) | 3.27(0.77,6.35) | 6.34(0.00,14.45) | 5.87(1.49,11.30) | 1.72(0.70,3.16) | 2.94(0.01,13.18) |
| Caribbean | 5.65(3.38,8.11) | 1.91(1.04,2.75) | 2.80(1.42,4.38) | 0.47(0.12,0.82) | 0.25(0.00,0.55) | 0.17(0.04,0.30) | 0.14(0.07,0.23) | 0.82(0.01,2.93) | 141.05(87.18,201.24) | 50.08(28.01,73.85) | 70.99(36.04,110.78) | 11.42(2.87,20.49) | 6.27(0.00,13.92) | 4.55(1.24,7.88) | 4.14(1.98,6.65) | 17.58(0.11,64.55) |
| Central Asia | 1.75(0.87,2.84) | 0.88(0.38,1.42) | 0.19(0.07,0.35) | 0.11(0.03,0.23) | 0.12(0.00,0.29) | 0.11(0.03,0.22) | 0.03(0.01,0.05) | 0.45(0.02,1.43) | 68.65(36.41,106.02) | 36.75(18.50,55.36) | 6.61(2.88,12.47) | 4.68(1.07,9.51) | 5.24(0.00,12.39) | 5.09(1.20,10.32) | 1.41(0.57,2.74) | 15.45(0.71,48.16) |
| Central Europe | 2.30(1.22,3.60) | 0.77(0.37,1.19) | 0.37(0.17,0.64) | 0.13(0.03,0.24) | 0.14(0.00,0.33) | 0.15(0.04,0.28) | 0.06(0.03,0.11) | 1.06(0.24,2.33) | 59.87(32.57,91.84) | 20.92(10.40,31.59) | 8.44(3.83,14.83) | 3.71(0.91,7.12) | 4.67(0.00,10.56) | 4.97(1.27,9.56) | 2.21(0.93,4.04) | 24.74(5.64,54.82) |
| Central Latin America | 8.91(5.06,13.03) | 2.98(1.54,4.47) | 4.01(1.95,6.28) | 0.48(0.12,0.90) | 0.46(0.00,1.06) | 0.25(0.06,0.47) | 0.30(0.13,0.53) | 1.96(0.12,5.75) | 224.86(129.21,327.66) | 76.50(40.12,116.35) | 98.84(48.92,160.47) | 13.13(3.39,24.63) | 13.31(0.00,30.28) | 7.55(1.89,14.55) | 9.50(4.05,17.62) | 45.42(2.82,135.05) |
| Central sub-Saharan Africa | 10.24(5.54,15.96) | 4.86(2.44,7.72) | 5.46(2.58,9.41) | 0.61(0.17,1.26) | 0.15(0.00,0.35) | 0.29(0.07,0.58) | 0.06(0.02,0.11) | 0.66(0.00,3.39) | 229.23(128.39,350.76) | 110.24(56.09,174.19) | 121.22(57.80,207.44) | 14.84(3.84,30.49) | 3.82(0.00,8.68) | 7.57(1.84,15.23) | 1.64(0.67,2.99) | 12.66(0.01,66.26) |
| East Asia | 2.11(1.08,3.28) | 0.75(0.38,1.16) | 0.12(0.05,0.23) | 0.26(0.07,0.49) | 0.28(0.00,0.62) | 0.08(0.02,0.14) | 0.03(0.01,0.05) | 0.82(0.17,1.96) | 48.70(25.79,74.68) | 16.43(8.68,25.95) | 2.04(0.83,4.12) | 5.70(1.40,11.29) | 6.81(0.00,14.53) | 1.87(0.46,3.42) | 0.74(0.33,1.29) | 20.15(4.60,44.72) |
| Eastern Europe | 1.04(0.57,1.57) | 0.45(0.22,0.69) | 0.29(0.13,0.48) | 0.06(0.01,0.12) | 0.06(0.00,0.14) | 0.14(0.04,0.29) | 0.01(0.01,0.03) | 0.17(0.01,0.55) | 38.27(21.62,56.27) | 17.50(8.65,26.62) | 10.05(4.66,17.17) | 2.30(0.55,4.78) | 2.25(0.00,5.61) | 5.18(1.25,10.85) | 0.54(0.21,1.06) | 6.13(0.33,19.36) |
| Eastern Sub-Saharan Africa | 9.07(5.08,13.90) | 3.92(2.00,6.03) | 4.38(2.13,7.13) | 0.44(0.10,0.85) | 0.14(0.00,0.33) | 0.19(0.05,0.36) | 0.04(0.02,0.08) | 1.90(0.09,5.61) | 186.90(105.39,283.60) | 82.38(42.09,127.06) | 91.58(44.70,149.34) | 8.54(2.00,16.34) | 2.96(0.00,7.01) | 3.96(0.98,7.65) | 0.97(0.43,1.73) | 35.93(1.72,107.14) |
| High-income Asia Pacific | 2.16(1.17,3.32) | 0.92(0.49,1.42) | 0.33(0.15,0.61) | 0.19(0.05,0.36) | 0.11(0.00,0.26) | 0.21(0.06,0.39) | 0.06(0.03,0.10) | 0.68(0.07,1.78) | 46.81(25.77,70.53) | 20.06(10.37,29.65) | 6.44(2.92,11.65) | 3.73(0.92,7.07) | 2.39(0.00,5.59) | 5.56(1.43,10.33) | 1.24(0.58,2.12) | 14.52(1.49,36.44) |
| High-income North America | 5.43(3.29,7.44) | 1.84(0.96,2.75) | 1.57(0.76,2.55) | 0.35(0.09,0.65) | 0.45(0.00,0.99) | 1.18(0.34,2.02) | 0.26(0.13,0.42) | 0.68(0.01,2.45) | 125.15(76.36,172.48) | 40.42(21.14,58.49) | 31.83(15.61,51.27) | 8.47(2.26,15.23) | 11.78(0.00,25.00) | 32.81(9.12,58.06) | 7.82(3.75,13.19) | 14.98(0.29,51.40) |
| North Africa and Middle East | 4.11(2.28,6.09) | 1.91(0.95,2.96) | 1.29(0.59,2.23) | 0.49(0.12,0.95) | 0.19(0.00,0.44) | 0.12(0.03,0.23) | 0.12(0.05,0.21) | 0.33(0.00,1.88) | 87.79(49.91,130.38) | 40.92(20.80,63.09) | 25.98(12.19,44.76) | 10.96(2.76,21.13) | 4.38(0.00,9.99) | 2.96(0.73,5.54) | 3.34(1.47,5.64) | 7.13(0.00,39.56) |
| Oceania | 2.56(1.37,3.96) | 0.88(0.44,1.46) | 0.73(0.34,1.31) | 0.45(0.12,0.82) | 0.27(0.00,0.61) | 0.11(0.03,0.20) | 0.09(0.04,0.14) | 0.36(0.03,1.05) | 64.85(35.61,99.93) | 23.18(12.17,38.27) | 19.63(9.61,34.25) | 10.59(2.77,19.72) | 6.53(0.00,14.99) | 2.63(0.66,4.80) | 2.08(0.95,3.48) | 8.71(0.73,25.60) |
| South Asia | 3.67(1.96,5.62) | 2.05(1.05,3.13) | 1.47(0.73,2.36) | 0.20(0.05,0.39) | 0.04(0.00,0.09) | 0.07(0.02,0.12) | 0.03(0.01,0.06) | 0.57(0.02,1.89) | 105.16(58.46,160.45) | 59.31(30.30,90.71) | 41.97(20.30,69.06) | 5.94(1.46,11.80) | 1.07(0.00,2.55) | 1.98(0.49,3.84) | 1.09(0.45,1.94) | 16.45(0.74,52.26) |
| Southeast Asia | 6.78(3.86,9.95) | 2.50(1.27,3.79) | 2.80(1.31,4.49) | 0.22(0.06,0.43) | 0.12(0.00,0.27) | 0.06(0.01,0.11) | 0.05(0.03,0.09) | 2.30(0.28,5.76) | 157.82(88.52,235.07) | 59.63(30.58,91.65) | 64.39(30.35,104.42) | 5.42(1.34,10.61) | 2.88(0.00,6.62) | 1.55(0.36,2.85) | 1.35(0.61,2.32) | 51.39(6.78,126.51) |
| Southern Latin America | 5.19(2.94,7.65) | 1.48(0.74,2.25) | 1.79(0.87,2.95) | 0.40(0.11,0.77) | 0.64(0.00,1.39) | 0.41(0.11,0.76) | 0.25(0.12,0.44) | 1.07(0.02,3.34) | 101.68(58.55,148.07) | 28.37(14.09,43.32) | 33.47(16.05,54.64) | 8.21(2.01,15.34) | 13.96(0.00,30.62) | 9.33(2.39,17.74) | 6.07(2.72,10.68) | 19.39(0.46,59.44) |
| Southern sub-Saharan Africa | 8.49(4.72,12.24) | 4.57(2.29,7.07) | 4.01(1.90,6.44) | 0.27(0.07,0.55) | 0.26(0.00,0.58) | 0.13(0.03,0.26) | 0.10(0.04,0.18) | 0.60(0.00,2.80) | 196.76(110.55,283.63) | 105.62(53.24,162.12) | 90.96(41.84,147.36) | 7.42(1.83,15.15) | 7.28(0.00,15.94) | 3.99(1.00,7.72) | 3.20(1.37,5.81) | 13.32(0.02,60.31) |
| Tropical Latin America | 5.16(3.22,7.07) | 1.38(0.79,1.99) | 2.32(1.26,3.54) | 0.37(0.09,0.68) | 0.59(0.00,1.27) | 0.20(0.05,0.37) | 0.17(0.08,0.27) | 1.09(0.04,3.22) | 124.37(77.61,168.66) | 33.40(19.36,47.81) | 55.15(30.19,84.13) | 9.12(2.34,16.98) | 15.82(0.00,33.53) | 5.62(1.43,10.32) | 4.74(2.18,7.96) | 23.91(0.88,69.76) |
| Western Europe | 2.09(1.21,3.11) | 0.72(0.35,1.13) | 0.69(0.32,1.12) | 0.15(0.04,0.29) | 0.18(0.00,0.42) | 0.30(0.08,0.59) | 0.06(0.03,0.11) | 0.28(0.00,1.05) | 44.32(25.37,64.23) | 15.25(7.34,22.92) | 13.56(6.33,22.29) | 3.06(0.75,5.93) | 3.97(0.00,9.16) | 7.69(1.90,15.10) | 1.62(0.71,2.94) | 5.83(0.11,20.98) |
| Western sub-Saharan Africa | 8.17(4.44,12.31) | 3.78(1.84,5.84) | 3.88(1.82,6.31) | 0.38(0.10,0.73) | 0.14(0.00,0.33) | 0.28(0.07,0.53) | 0.03(0.01,0.06) | 1.00(0.00,4.02) | 174.29(96.00,261.75) | 81.41(40.34,125.28) | 81.46(37.26,133.46) | 9.10(2.31,17.17) | 3.42(0.00,8.21) | 7.41(1.86,14.58) | 0.87(0.35,1.66) | 19.33(0.05,76.58) |
